# Supplementary material for: In-situ electro-responsive through-space coupling enabling foldamers as volatile memory elements
Source: Nat Commun. 2023 Oct 6;14:6250. doi: 10.1038/s41467-023-42028-5 (PMC10558558; doi:10.1038/s41467-023-42028-5)
Supplement: Supplementary file 1 — Supplementary Information [file 41467_2023_42028_MOESM1_ESM.pdf]

SUPPLEMENTARY INFORMATION

In-situ electro-responsive through-space coupling enabling foldamers as volatile memory elements

Jinshi Li,[1] Pingchuan Shen,[1] Zeyan Zhuang,[1] Junqi Wu,[1] Ben Zhong Tang[2] and Zujin Zhao[1,*]

[1] State Key Laboratory of Luminescent Materials and Devices, Guangdong Provincial Key Laboratory of Luminescence from Molecular Aggregates, South China University of Technology, Guangzhou, 510640, China. Email: mszjzhao@scut.edu.cn.

[2] School of Science and Engineering, Shenzhen Institute of Aggregate Science and Technology, The Chinese University of Hong Kong, Shenzhen, Guangdong 518172, China.

Contents

Supplementary Note 1. Experimental details S1
    Syntheses and characterization S1
Supplementary Note 2. Conductance measurement based on STM-BJ S9
    Supplementary Note 2.1 Conductance measurement for *f*-Fu and *f*-Th under positive biases. S9
    Supplementary Note 2.2 Conductance measurement for *f*-Fu and *f*-Th under negative biases. S11
    Supplementary Note 2.3 Conductance measurement under variable piezo rates for mechanical analysis. S13
    Supplementary Note 2.4 Further discussion on control molecules S14
    Supplementary Note 2.5 Further discussion of the existence of intermolecular through-space interaction during junction formation S19
Supplementary Note 3. Theoretica lcalculation S22
Supplementary Note 4. Electrochemical gating experiment S30
Supplementary Note 5. NMR spectra S36
Supplementary Note 6. References S44

Supplementary Note 1. Experimental details

Syntheses and characterization

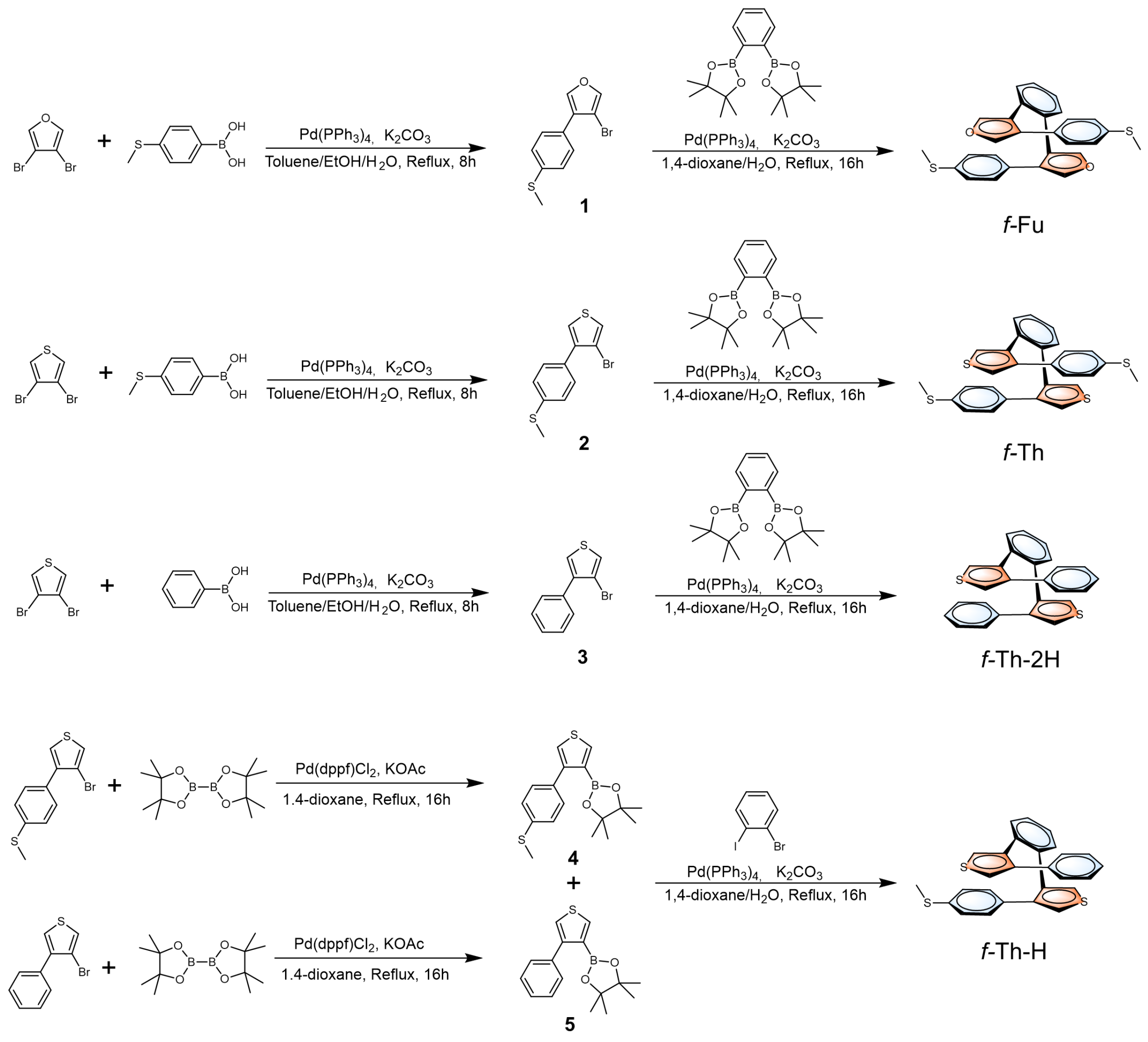

Supplementary Figure 1 Synthetic routes of the folded molecules studied in this work.

3-Bromo-4-(4-(methylthio)phenyl)furan (1): Into 250 mL two-necked round bottom flask was placed 3,4-dibromofuran (2.93 g, 13 mmol), (4-(methylthio)phenyl)boronic acid (1.68 g, 10 mmol), $Pd(PPh_3)_4$ (578 mg, 0.5 mmol) and K_2CO_3 (2.07 g, 15 mmol). The flask was evacuated under vacuum and flushed with dry nitrogen three times and then 100 mL mixture of toluene, ethyl alcohol and deionized water (3/1/1, *v*/*v*/*v*) was added. The reaction mixture was heated and refluxed at 95 ^oC for 8 h. After cooling to room temperature, the mixture was poured into water and extracted with dichloromethane three times. The combined organic layers were dried over anhydrous magnesium sulfate. After filtration and solvent evaporation, the crude product was purified by silica-gel column chromatography with petroleum ether as eluent. Product **1** as colorless transparent liquid was obtained in 76% yield. ^1H NMR (500 MHz, CD_2Cl_2), δ (ppm): 7.90 (d, J = 2.0 Hz, 1H), 7.57 (d, J = 2.0 Hz, 1H), 7.51–7.44 (m, 2H), 7.43 – 7.36 (m, 2H), 2.50 (s, 3H). ^{13}C NMR (125 MHz, $(CD_3)_2SO$), δ (ppm): 144.41, 141.67, 138.42, 128.60, 126.99, 126.49, 125.38, 100.62, 15.08. HRMS (MALDI-TOF): *m/z* [M^+] calcd. $C_{11}H_9BrOS$, 267.9557; found, 267.9579.

3-Bromo-4-(4-(methylthio)phenyl)thiophene (2): The synthetic procedure was analogous to that described for compound **1**, using 3,4-dibromothiophene (3.12 mg, 13 mmol), (4-(methylthio)phenyl)boronic acid (1.68 g, 10 mmol), $Pd(PPh_3)_4$ (578 mg, 0.5 mmol) and K_2CO_3 (2.07 g, 15 mmol). Product **2** as colorless transparent crystal was

obtained in 80% yield. ^{1}H NMR (500 MHz, CD_2Cl_2), δ (ppm): 7.44–7.41 (m, 2H), 7.39 (d, J = 7.5 Hz, 1H), 7.31 – 7.27 (m, 3H), 2.51 (s, 3H). ^{13}C NMR (125 MHz, $(CD_3)_2SO$), δ (ppm): 142.01, 139.27, 132.43, 130.03, 126.58, 125.00, 124.13, 112.02, 16.04. HRMS (MALDI-TOF): *m/z* [M^+] calcd. $C_{11}H_9BrS_2$, 283.9329; found, 283.9339.

3-Bromo-4-phenylthiophene (3): The synthetic procedure was analogous to that described for compound **1**, using 3,4-dibromothiophene (3.12 mg, 13 mmol), (4-(methylthio)phenyl)boronic acid (1.68 g, 10 mmol), $Pd(PPh_3)_4$ (578 mg, 0.5 mmol) and K_2CO_3 (2.07 g, 15 mmol). Product **3** as colorless transparent crystal was obtained in 80% yield. ^{1}H NMR (500 MHz, CD_2Cl_2), δ (ppm): 7.52–7.47 (m, 2H), 7.44 –7.34 (m, 4H) 7.28 (d, J = 5.0 Hz, 1H). ^{13}C NMR (125 MHz, $(CD_3)_2SO$), δ (ppm): 143.19, 136.47, 130.29, 129.48, 129.07, 125.53, 124.99, 112.13. HRMS (MALDI-TOF): *m/z* [M^+] calcd. $C_{10}H_7BrS_2$, 237.9452; found, 237.9459.

4,4,5,5-Tetramethyl-2-(4-(4-(methylthio)phenyl)thiophen-3-yl)-1,3,2-dioxaborolane (4): Into 250 mL two-necked round bottom flask was placed **2** (1.70 g, 6 mmol), bis(pinacolato)diboron (2.54 g, 10 mmol), $Pd(dppf)_2Cl_2$ (183 mg, 0.25 mmol) and KOAc (1.47 g, 15 mmol). The flask was evacuated under vacuum and flushed with dry nitrogen three times and then 90 mL 1,4-dioxane was added. The reaction mixture was heated and refluxed at 120 °C for 16 h. After cooling to room temperature, the mixture was poured into water and extracted with dichloromethane three times. The combined organic layers were dried over anhydrous magnesium sulfate. After filtration and solvent evaporation, the crude product was purified by silica-gel column chromatography with mixture of petroleum ether and dichloromethane as eluent. Product **4** as light-yellow crystal was obtained in 90% yield. ^{1}H NMR (500 MHz, $CDCl_3$), δ (ppm): 7.91 (d, J = 2.5 Hz, 1H), 7.56–7.53 (m, 1H), 7.44–7.39 (m, 2H), 7.31–7.22 (m, 2H), 2.51 (s, 3H), 1.26 (s, 12H). ^{13}C NMR (125 MHz, $CDCl_3$), δ (ppm): 147.58, 139.35, 138.00, 133.94, 129.97, 127.43, 127.38, 126.53, 124.18, 120.54, 26.11, 16.26. HRMS (MALDI-TOF): *m/z* [M^+] calcd. $C_{17}H_{21}BO_2S_2$, 332.1076; found, 332.1094.

4,4,5,5-Tetramethyl-2-(4-phenylthiophen-3-yl)-1,3,2-dioxaborolane (5): The synthetic procedure was analogous to that described for compound **4**, using 3 (1.42 g, 6 mmol), (4-(methylthio)phenyl)boronic acid (1.68 g, 10 mmol), $Pd(PPh_3)_4$ (578 mg, 0.5 mmol) and K_2CO_3 (2.07 g, 15 mmol). Product **5** as colorless transparent crystal was obtained in 95% yield. ^{1}H NMR (500 MHz, CD_2Cl_2), δ (ppm): 7.91 (d, J = 3.5 Hz, 1H), 7.49–7.46 (m, 2H), 7.51–7.44 (m, 2H), 7.38–7.33 (m, 2H), 7.33–7.27 (m, 2H), 1.27 (s, 12H). ^{13}C NMR (125 MHz, $(CD_3)_2SO$), δ (ppm): 148.96, 139.05, 138.46, 130.20, 129.14, 128.31, 124.56, 84.97, 25.77. HRMS (MALDI-TOF): *m/z* [M^+] calcd. $C_{16}H_{19}BO_2S$, 286.1199; found, 286.1208.

1,2-Bis(4-(4-(methylthio)phenyl)furan-3-yl)benzene (*f*-Fu): The synthetic procedure was analogous to that described for compound **1**, using compound **1** (801 mg, 3 mmol), 1,2-bis(4,4,5,5-tetramethyl-1,3,2-dioxaborolan-2-yl)benzene (330 mg, 1 mmol), $Pd(PPh_3)_4$ (57 mg, 0.05 mmol) and K_2CO_3 (554mg, 4 mmol). Product *f*-Fu as light-yellow solid was obtained in 40% yield. ^{1}H NMR (500 MHz, CD_2Cl_2), δ (ppm): 7.33 (d, J = 2.5 Hz, 2H), 7.32–7.29 (m, 2H), 7.27–7.22 (m, 2H), 7.01 (d, J = 10.0 Hz, 4H), 6.81 (d, J = 2.5 Hz, 2H), 6.74 (d, J = 10.5 Hz, 4H), 2.39 (s, 6H). ^{13}C NMR (125 MHz, CD_2Cl_2), δ (ppm): 141.63, 138.89, 136.84, 131.75, 131.14, 129.20, 127.78, 127.55, 126.21, 126.11, 124.70, 16.44. HRMS (MALDI-TOF): *m/z* [M^+] calcd. $C_{28}H_{22}O_2S_2$, 454.1061; found, 454.1010.

1,2-Bis(4-(4-(methylthio)phenyl)thiophen-3-yl)benzene (*f*-Th): The synthetic procedure was analogous to that described for compound **1**, using compound **2** (852 mg, 3 mmol) 1,2-bis(4,4,5,5-tetramethyl-1,3,2-dioxaborolan-2-yl)benzene (330 mg, 1 mmol), $Pd(PPh_3)_4$ (57 mg, 0.05 mmol) and K_2CO_3 (554 mg, 4 mmol). Product *f*-Th as yellow solid was obtained in 46% yield. ^{1}H NMR (500 MHz, CD_2Cl_2), δ (ppm): 7.33–7.28 (m, 2H), 7.25–7.20 (m, 2H), 7.00–6.94 (m, 6H), 6.59 (d, J = 10.0 Hz, 4H), 6.46 (d, J = 4 Hz, 2H), 2.41 (s, 6H). ^{13}C NMR (125 MHz, CD_2Cl_2), δ (ppm): 141.61, 140.59, 136.67, 133.45, 131.30, 128.26, 127.34, 125.97, 125.25, 121.76, 15.22. HRMS (MALDI-TOF): *m/z* [M^+] calcd. $C_{28}H_{22}S_4$, 486.0604; found, 486.0612.

1,2-Bis(4-phenylthiophen-3-yl)benzene (*f*-Th-2H): The synthetic procedure was analogous to that described for compound **1**, using compound **3** (714 mg, 3 mmol), 1,2-bis(4,4,5,5-tetramethyl-1,3,2-dioxaborolan-2-yl)benzene (330 mg, 1 mmol), $Pd(PPh_3)_4$ (57 mg, 0.05 mmol) and K_2CO_3 (554 mg, 4 mmol). Product *f*-Th-2H as yellow solid

was obtained in 40% yield. ^{1}H NMR (500 MHz, CD_2Cl_2), δ (ppm): 7.33–7.27 (m, 2H), 7.24–7.19 (m, 2H), 7.14–7.04 (m, 6H), 6.99 (d, J = 4.0 Hz, 4H), 6.71–6.66 (m, 4H), 6.35 (d, J = 5.0 Hz, 4H). ^{13}C NMR (125 MHz, CD_2Cl_2), δ (ppm): 142.20, 140.68, 136.62, 136.12, 131.29, 127.85, 127.76, 127.28, 126.40, 125.10, 121.85. HRMS (MALDI-TOF): *m/z* [M^+] calcd. $C_{28}H_{22}O_2S_2$, 394.0850; found, 394.0857.

1,2-Bis(4-phenylthiophen-3-yl)benzene (*f*-Th-H): Into 250 mL two-necked round bottom flask was placed 1-bromo-2-iodobenzene (282 mg, 1 mmol), **5** (343 mg, 1.2 mmol), $Pd(PPh_3)_4$ (57 mg, 0.05 mmol) and K_2CO_3 (552 mg, 4 mmol). The flask was evacuated under vacuum and flushed with dry nitrogen three times and then 100 mL mixture of toluene, ethyl alcohol and deionized water (3/1/1, *v*/*v*/*v*) was added. The reaction mixture was heated and refluxed at 95 °C for 8 h. After monitoring the reaction with the disappearance of 1-bromo-2-iodobenzene, compound **4** (664 mg, 2 mmol) was added into the mixture to further react for 8 h. After cooling to room temperature, the mixture was poured into water and extracted with dichloromethane three times. The combined organic layers were dried over anhydrous magnesium sulfate. After filtration and solvent evaporation, the crude product was purified by silica-gel column chromatography with petroleum ether as eluent. Product *f*-Th-H as yellow solid was obtained in 34% yield. ^{1}H NMR (500 MHz, CD_2Cl_2), δ (ppm): 7.32–7.28 (m, 2H), 7.25–7.19 (m, 2H), 7.14–7.04 (m, 3H), 7.00 (d, J = 5.0 Hz, 1H), 6.98–6.96 (m, 2H), 6.96–6.94 (m, 1H), 6.70–6.66 (m, 2H), 6.62–6.58 (m, 2H), 6.44 (d, J = 5.0 Hz, 1H), 6.37 (d, J = 5.0 Hz, 1H), 2.41 (s, 3H). ^{13}C NMR (125 MHz, CD_2Cl_2), δ (ppm): 143.55, 142.89, 141.98, 141.92, 137.93, 137.90, 137.38, 137.31, 135.26, 132.64, 132.58, 129.58, 129.15, 129.08, 128.63, 127.75, 127.30, 126.48, 123.26, 122.97, 16.98. HRMS (MALDI-TOF): *m/z* [M^+] calcd. $C_{28}H_{22}O_2S_2$, 440.0727; found, 440.0734.

4,4''''-Bis(methylthio)-1,1':2',1'':2'',1''':2''',1''''-quinquephenyl (*f*-Ph): The synthesis of *f*-Ph follows the previous reported procedure.[1] ^{1}H NMR (500 MHz, CD_2Cl_2), δ (ppm): 7.40–7.35 (m, 2H), 7.23–7.15 (m, 2H), 7.17–7.05 (m, 4H), 6.99 (d, J = 7.0 Hz, 2H), 6.94–6.88 (m, 6H), 6.52 (d, J = 8.0 Hz, 2H), 6.16 (d, J = 8.0 Hz, 2H). ^{13}C NMR (125 MHz, CD_2Cl_2), δ (ppm): 141.94, 140.69, 140.65, 139.37, 137.47, 133.73, 132.91, 131.57, 130.75, 130.22, 128.61, 138.38, 128.05, 127.54, 127.19, 126.84, 17.12. HRMS (MALDI-TOF): *m/z* [M^+] calcd. $C_{32}H_{26}S_2$, 474.1476; found, 474.1478).

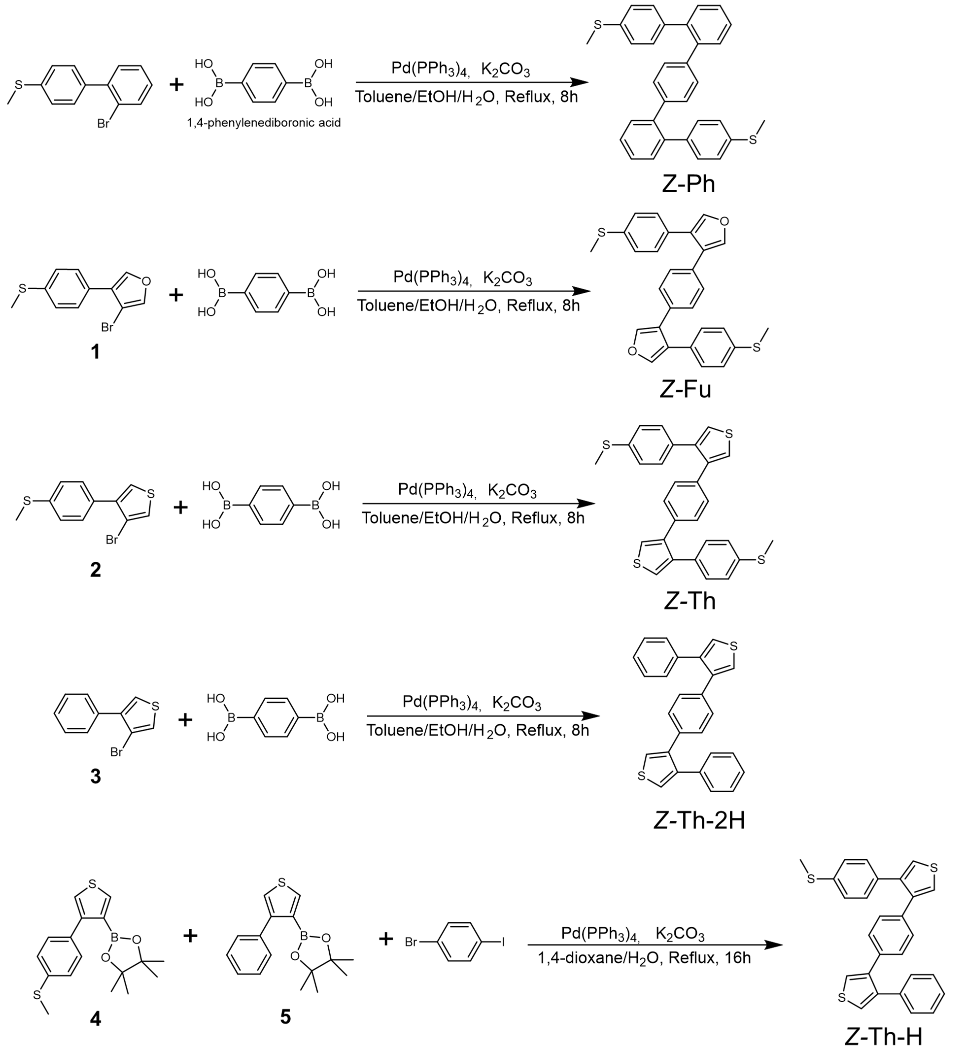

Supplementary Figure 2 Synthetic routes of the *Z*-shaped non-stacked molecules studied in this work.

1,4-Bis(4-(4-(methylthio)phenyl)furan-3-yl)benzene (*Z*-Fu): The synthetic procedure was analogous to that described for *f*-Fu with compound **1** (804 mg, 3 mmol), 1,4-phenylenediboronic acid (166 mg, 1 mmol), $Pd(PPh_3)_4$ (116 mg, 0.1 mmol) and K_2CO_3 (552 mg, 4 mmol). The obtained product was further purified by recrystallization in the mixture of *n*-hexane and dichloromethane. White solid of *Z*-Fu was obtained in 44% yield. ^{1}H NMR (500 MHz, CD_2Cl_2), δ (ppm): 7.58 (d, J = 10.0 Hz, 4H), 7.19–7.15 (m, 12H), 2.47 (s, 6H). ^{13}C NMR (125 MHz, CD_2Cl_2), δ (ppm): 140.79, 140.76, 137.51, 128.92, 128.62, 128.48, 126.59, 125.49, 124.48, 12.43. HRMS (MALDI-TOF): *m/z* [M^+] calcd. $C_{28}H_{22}O_2S_2$, 454.1061; found, 454.1010.

1,4-Bis(4-(4-(methylthio)phenyl)furan-3-yl)benzene (*Z*-Th): The synthetic procedure was analogous to that described for *f*-Fu with compound **1** (804 mg, 3 mmol), 1,4-phenylenediboronic acid (166 mg, 1 mmol), $Pd(PPh_3)_4$ (116 mg, 0.1 mmol) and K_2CO_3 (552 mg, 4 mmol). The obtained product was further purified by recrystallization in the mixture of *n*-hexane and dichloromethane. White solid of *Z*-Fu was obtained in 52% yield. ^{1}H NMR (500 MHz, CD_2Cl_2), δ (ppm): 7.34 (d, J = 10.0 Hz, 4H), 7.17–7.07 (m, 12H), 2.48 (s, 6H). ^{13}C NMR (125 MHz, CD_2Cl_2), δ (ppm): 129.33, 128.78, 125.90, 124.09, 123.93, 15.41. HRMS (MALDI-TOF): *m/z* [M^+] calcd. $C_{28}H_{22}S_4$, 486.0604; found, 486.0612.

1,2-Bis(4-phenylthiophen-3-yl)benzene (*Z*-Th-2H): The synthetic procedure was analogous to that described for

compound **1**, using compound **3** (714 mg, 3 mmol), 1,4-phenylenediboronic acid (166 mg, 1 mmol), $Pd(PPh_3)_4$ (57 mg, 0.05 mmol) and K_2CO_3 (554 mg, 4 mmol). Product *Z*-Th-2H as white solid was obtained in 67% yield. ^{1}H NMR (500 MHz, CD_2Cl_2), δ (ppm): 7.36–7.32 (m, 4H), 7.28–7.23 (m, 6H), 7.21–7.17 (m, 4H), 7.08 (s, 4H). ^{13}C NMR (125 MHz, CD_2Cl_2), δ (ppm): 141.74, 141.33, 136.56, 135.15, 128.98, 128.73, 128.09, 126.87, 124.13, 123.96. HRMS (MALDI-TOF): *m/z* [M^+] calcd. $C_{28}H_{22}O_2S_2$, 394.0850; found, 394.0857.

1,2-Bis(4-phenylthiophen-3-yl)benzene (*Z*-Th-H): The synthetic procedure was analogous to that described for ***f*-Th-H**, using 1-bromo-2-iodobenzene (282 mg, 1mmol), compound **5** (343 mg, 1.2 mmol), $Pd(PPh_3)_4$ (57 mg, 0.05 mmol), compound **4** (664 mg, 2 mmol) and K_2CO_3 (554 mg, 4 mmol). Product *Z*-Th-H as white solid was obtained in 60% yield. ^{1}H NMR (500 MHz, CD_2Cl_2), δ (ppm): 7.38–7.30 (m, 4H), 7.28–7.24 (m, 3H), 7.22–7.17 (m, 2H), 7.10–7.08 (m, 4H), 2.47 (s, 3H). ^{13}C NMR (125 MHz, CD_2Cl_2), δ (ppm): 141.75, 141.32, 141.25, 141.10, 137.28, 136.58, 135.18, 135.08, 133.20, 129.33, 129.00, 128.78, 128.73, 128.09, 126.87, 125.92, 124.15, 142.02, 132.96, 123.86, 15.38. HRMS (MALDI-TOF): *m/z* [M^+] calcd. $C_{28}H_{22}O_2S_2$, 440.0727; found, 440.0734.

4,4''''-Bis(methylthio)-1,1':2',1'':4'',1''':2''',1''''-quinquephenyl (*Z*-Ph): The synthetic procedure was analogous to that described for compound **1** with (2'-bromo-[1,1'-biphenyl]-4-yl)(methyl)sulfane (834 mg, 3 mmol), 1,4-phenylenediboronic acid (166 mg, 1 mmol), $Pd(PPh_3)_4$ (116 mg, 0.1 mmol) and K_2CO_3 (552 mg, 4 mmol). The obtained product was further purified by recrystallization in the mixture of *n*-hexane and dichloromethane. White solid of *Z*-Ph was obtained in 65% yield. ^{1}H NMR (500 MHz, $CDCl_3$), δ (ppm): 7.43–7.36 (m, 8H), 7.10 (d, J = 5.0 Hz, 4H), 7.05 (d, J = 7.5 Hz, 4H), 7.02 (s, 4H), 2.47 (s, 6H). ^{13}C NMR (125 MHz, $CDCl_3$), δ (ppm): 140.10, 139.79, 139.59, 138.24, 136.68, 130.60, 130.50, 130.30, 129.48, 127.53, 127.44, 125.89, 15.79. HRMS (MALDI-TOF): *m/z* [M^+] calcd. $C_{32}H_{26}S_2$, 474.1476; found, 474.1457.

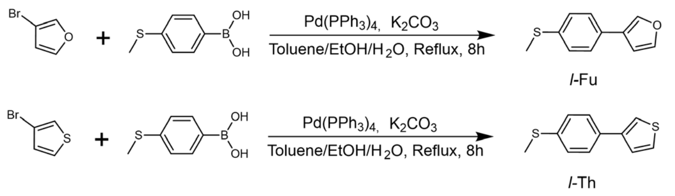

Supplementary Figure 3 Synthetic routes of the linear monomer studied in this work.

3-(4-(Methylthio)phenyl)furan (*l*-Fu): The synthetic procedure was analogous to that described for compound **1** with 3-bromofuran (145 mg, 1 mmol), (4-(methylthio)phenyl)boronic acid (252 mg, 1.5 mmol), $Pd(PPh_3)_4$ (57 mg, 0.05 mmol) and K_2CO_3 (276 mg, 2 mmol). The obtained product was further purified by recrystallization in the mixture of *n*-hexane and dichloromethane. White solid of *l*-Fu was obtained in 92% yield. ^{1}H NMR (500 MHz, $CDCl_3$), δ (ppm): 7.71 (s, 1H), 7.48–7.45 (m, 1H), 7.41 (d, J = 7.5 Hz, 2H), 7.27 (d, J = 7.5 Hz, 2H), 6.68 (s, 1H), 2.50 (s, 3H). ^{13}C NMR (125 MHz, $CDCl_3$), δ (ppm): 143.70, 138.30, 136.97, 129.40, 127.17, 126.28, 125.95, 108.72, 16.06. HRMS (MALDI-TOF): *m/z* [M^+] calcd. $C_{11}H_{10}OS$, 190.0452; found, 190.0479.

3-(4-(Methylthio)phenyl)thiophene (*l*-Th): The synthetic procedure was analogous to that described for **1** with 3-bromothiophene (162 mg, 1 mmol), (4-(methylthio)phenyl)boronic acid (252 mg, 1.5 mmol), $Pd(PPh_3)_4$ (57 mg, 0.05 mmol) and K_2CO_3 (276 mg, 2 mmol). The obtained product was further purified by recrystallization in the mixture of *n*-hexane and dichloromethane. White solid of *l*-Th was obtained in 85% yield. ^{1}H NMR (500 MHz, $CDCl_3$), δ (ppm): 7.54 (d, J = 10 Hz, 1H), 7.47–7.45 (m, 1H), 7.42–7.38 (m, 2H), 7.28 (d, J = 10 Hz, 2H), 2.50 (s, 3H). ^{13}C NMR (125 MHz, $CDCl_3$), δ (ppm): 142.94,138.81,133.89,128.08, 128.01, 127.65, 127.34,121.18, 16.89. HRMS (MALDI-TOF): *m/z* [M^+] calcd. $C_{11}H_{10}S_2$, 206.0224; found, 206.0251.

Crystallography analysis

Crystal data for *f*-Fu (CCDC 2104355): $C_{28}H_{22}O_2S_2$, *M*w = 454.57, monoclinic, P21/c, a = 7.8983(10), b = 13.4864(2), c = 20.8307(3) Å, α = 90°, β = 92.966(10)°, γ = 90°, V = 2215.91(5) Å^3, Z = 4, Dc = 1.363 g cm^{-3}, μ = 0.2361 mm^{-1}, F(000) = 952, T = 150(10) K, R_1 (I > 2σ(I)) = 0.0328, wR_2 (I > 2σ(I)) = 0.0866, R_1 (all data) = 0.0354, wR_2 (all data) = 0.0881.

Crystal data for *f*-Th (CCDC 2205494): $C_{28}H_{22}S_{24}$, *M*w = 486.69, monoclinic, P21/n, a = 8.8176(3), b = 13.8213(5), c = 19.2644(7) Å, α = 90°, β = 90.6630(10)°, γ = 90°, V = 2347.61(14) Å^3, Z = 4, Dc = 1.377 g cm^{-3}, μ = 0.420 mm^{-1}, F(000) = 1016, T = 173(2) K, R_1 (I > 2σ(I)) = 0.0333, wR_2 (I > 2σ(I)) = 0.0806, R_1 (all data) = 0.0528, wR_2 (all data) = 0.0930.

Crystal data for *f*-Th-H (CCDC 2205495): $C_{27}H_{20}S_3$, *M*w = 440.61, monoclinic, Pbca, a = 15.5791(5), b = 8.3820(2), c = 33.1015(12) Å, α = 90°, β = 90°, γ = 90°, V = 4322.5(2) Å^3, Z = 8, Dc = 1.354 g cm^{-3}, μ = 0.355 mm^{-1}, F(000) = 1840, T = 173(2) K, R_1 (I > 2σ(I)) = 0.0420, wR_2 (I > 2σ(I)) = 0.1161, R_1 (all data) = 0.0621, wR_2 (all data) = 0.1052.

Crystal data for *f*-Th-2H (CCDC 2205496): $C_{26}H_{18}S_2$, *M*w = 394.52, triclinic, P-1, a = 8.8616(4), b = 9.0068(4), c = 13.3880(6) Å, α = 86.632(2)°, β = 89.590(2)°, γ = 70.052(2)°, V = 1002.62(8) Å^3, Z = 2, Dc = 1.307 g cm^{-3}, μ = 0.274 mm^{-1}, F(000) = 412, T = 173(2) K, R_1 (I > 2σ(I)) = 0.0351, wR_2 (I > 2σ(I)) = 0.0792, R_1 (all data) = 0.0511, wR_2 (all data) = 0.0890.

Crystal data for *f*-Ph (CCDC 2000283): $C_{32}H_{26}S_2$, *M*w = 474.65, triclinic, P-1, a = 9.6357(10), b = 10.6121(13), c = 12.6923(15) Å, α = 82.645(4)°, β = 78.544(4)°, γ = 77.796(4)°, V = 1238.2(2) Å^3, Z = 2, Dc = 1.273 g cm^{-3}, μ = 0.234 mm^{-1}, F(000) = 500, T = 171(2) K, R_1 (I > 2σ(I)) = 0.0657, wR_2 (I > 2σ(I)) = 0.0864, R_1 (all data) = 0.156, wR_2 (all data) = 0.1057.

Crystal data for *Z*-Fu (CCDC 2104356): $C_{28}H_{22}O_2S_2$, *M*w = 454.57, monoclinic, P21/n, a = 9.5492(7), b = 5.5687(3), c = 20.7756(15) Å, α = 90°, β = 91.375(3)°, γ = 90°, V = 1104.46(13) Å^3, Z = 2, Dc = 1.367 g cm^{-3}, μ = 0.265 mm^{-1}, F(000) = 476, T = 173(2) K, R_1 (I > 2σ(I)) = 0.0421, wR_2 (I > 2σ(I)) = 0.0858, R_1 (all data) = 0.0696, wR_2 (all data) = 0.098.

Crystal data for *Z*-Th (CCDC 2205497): $C_{28}H_{22}S_4$, *M*w = 486.69, triclinic, P-1, a = 5.7784(3), b = 10.1984(5), c = 11.2841(5) Å, α = 113.234(2)°, β = 95.388(2)°, γ = 101.849(2)°, V = 586.61(5) Å^3, Z = 1, Dc = 1.378 g cm^{-3}, μ = 0.420 mm^{-1}, F(000) = 254, T = 173(2) K, R_1 (I > 2σ(I)) = 0.0497, wR_2 (I > 2σ(I)) = 0.1129, R_1 (all data) = 0.0702, wR_2 (all data) = 0.1355.

Crystal data for *Z*-Th-2H (CCDC 2205498): $C_{26}H_{14}S_2$, *M*w = 394.52, monoclinic, P21/c, a = 6.5978(2), b = 10.8082(4), c = 13.6829(6) Å, α = 90°, β = 92.028(2)°, γ = 90°, V = 975.12(6) Å^3, Z = 4, Dc = 1.344 g cm^{-3}, μ = 0.282 mm^{-1}, F(000) = 412, T = 173(2) K, R_1 (I > 2σ(I)) = 0.0390, wR_2 (I > 2σ(I)) = 0.0883, R_1 (all data) = 0.0505, wR_2 (all data) = 0.1002.

Crystal data for *Z*-Ph (CCDC 2104357): $C_{32}H_{26}S_2$, *M*w = 474.65, triclinic, P-1, a = 5.6249(3), b = 10.5321(6), c = 11.6945(6) Å, α = 115.06(2)°, β = 92.851(2)°, γ = 102.749(2)°, V = 604.02(6) Å^3, Z = 1, Dc = 1.305 g cm^{-3}, μ = 0.240 mm^{-1}, F(000) = 250, T = 173(2) K, R_1 (I > 2σ(I)) = 0.0367, wR_2 (I > 2σ(I)) = 0.0837, R_1 (all data) = 0.0502, wR_2 (all data) = 0.970.

Crystal data for *l*-Th (CCDC 2284798): $C_{11}H_{10}S_2$, *M*w = 206.31, monoclinic, P 21/c, a = 5.5810(8), b = 7.7248(9), c = 23.012(3) Å, α = 90°, β = 91.524(4)°, γ = 90°, V = 991.8(2) Å^3, Z = 4, Dc = 1.382 g cm^{-3}, μ = 0.483 mm^{-1}, F(000) = 432, T = 173(2) K, R_1 (I > 2σ(I)) = 0.0579, wR_2 (I > 2σ(I)) = 0.0974, R_1 (all data) = 0.1125, wR_2 (all data) = 0.0844.

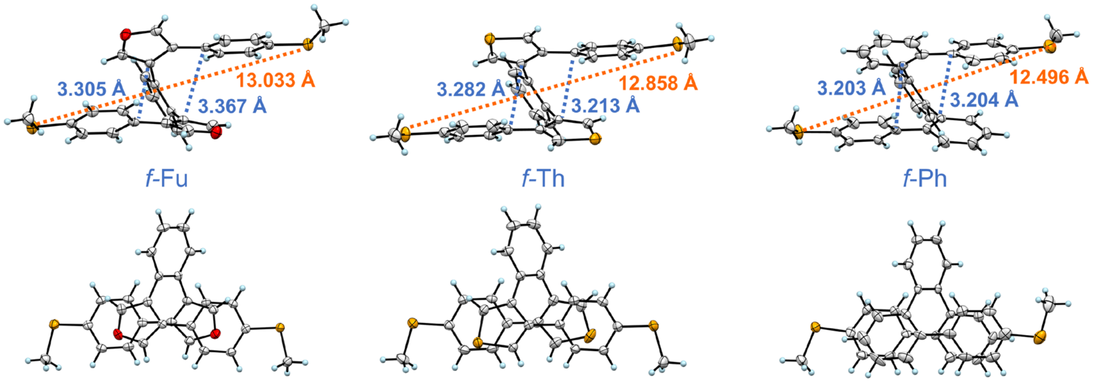

Supplementary Figure 4 Single crystal structures and structural parameters of *f*-Fu, *f*-Th and *f*-Ph.

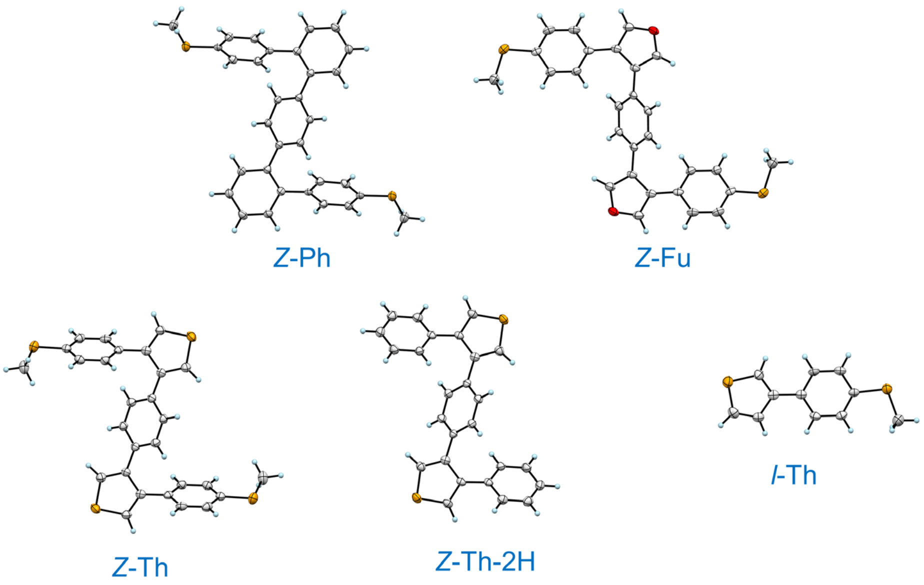

Supplementary Figure 5 Single crystal structures of *Z*-Fu, *Z*-Ph, *Z*-Th, *Z*-Th-2H and *l*-Th.

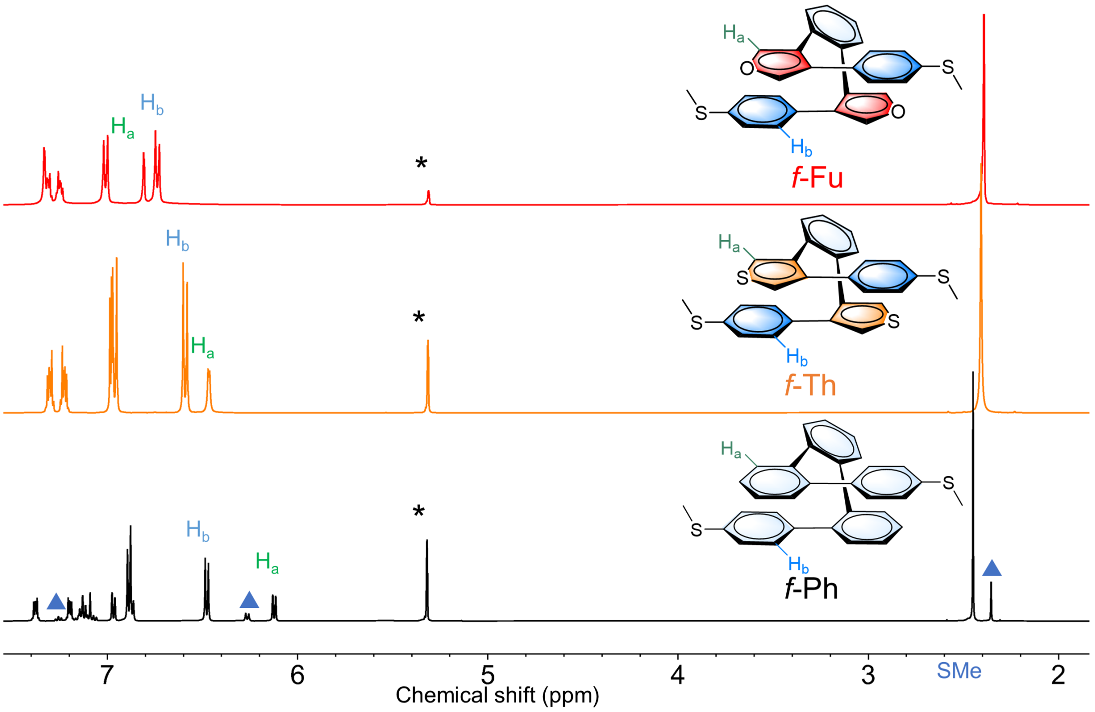

Supplementary Figure 6 ^{1}H NMR spectra in CD_2Cl_2 of *f*-Fu and *f*-Th at room temperature and *f*-Ph at –5 °C. The asterisks mark the signals of CD_2Cl_2 and the triangles label the minor conformer's signal of *f*-Ph.

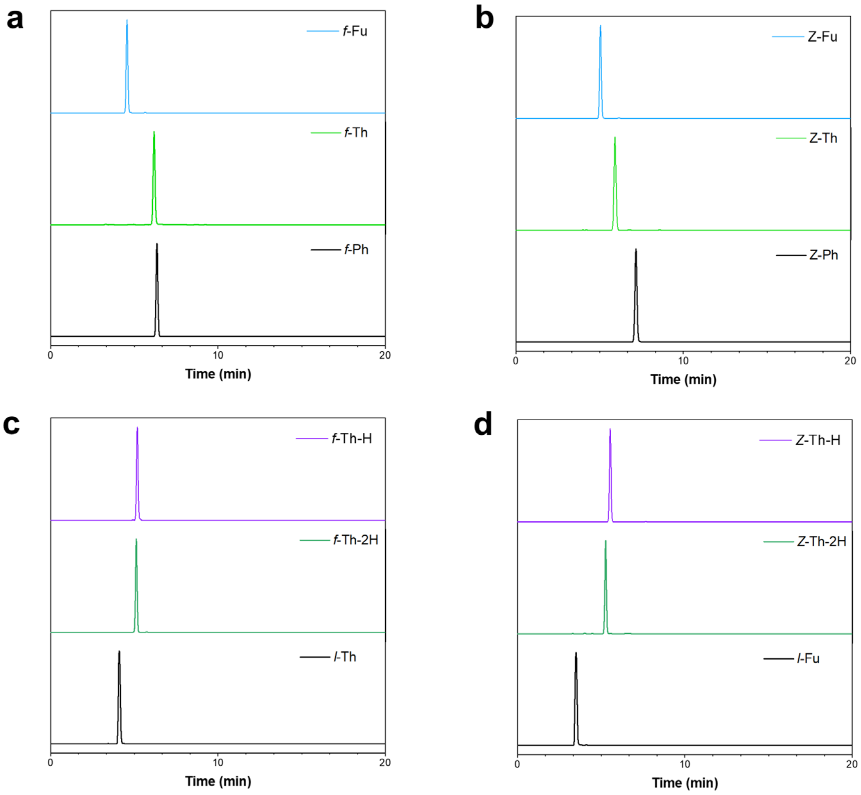

Supplementary Figure 7 Purity analysis of the studied molecules. The HPLC spectra of the studied molecules in MeCN detected at 254 nm.

Supplementary Note 2. Conductance measurement based on STM-BJ

Data analysis processes[2,3]

In the measurement of single-molecule conductance, thousands of individual single conductance-displacement traces were recorded in each experiment, and statistical approach is applied to determine the most probable conductance and the stretching distance. 1D conductance histograms were constructed by collecting individual traces with a bin size of 1100 for log(G/G_0) from −10 to +1, and 1000 for the distance between the tip and substrate (Δz) from −0.5 to 3 nm. The conductance distribution was extracted by calculating the data density in each bin. The peak in a conductance histogram was determined by Gaussian fitting, which represents the most probable molecular conductance. 2D conductance-displacement histograms were plotted by overlapping each individual trace with a bin size of 1100 for log(G/G_0) from −10 to +1, and 1000 for Δz from −0.5 to 3 nm. All traces were aligned with a relative zero point ($\Delta z = 0$) at $G = 0.5\ G_0$. Then, the 2D conductance distribution versus the relative distance was constructed by the data counts in each bin. To construct the displacement distribution histograms, firstly the relative stretching distance, Δz, was determined from the position where the conductance was 0.5 G_0 (after the rupture of the gold-gold atomic break at G_0), to the molecular conductance region, just before the end of molecular plateau. The peak represented the most probable plateau length. The junction formation probability was analyzed based on an auto-classification algorithm of the spectral clustering. The mean values and standard deviations of junction formation probability were statistically calculated from repeated measurements over three times to ensure the reliability of data.

Supplementary Note 2.1 Conductance measurement for *f*-Fu and *f*-Th under positive biases.

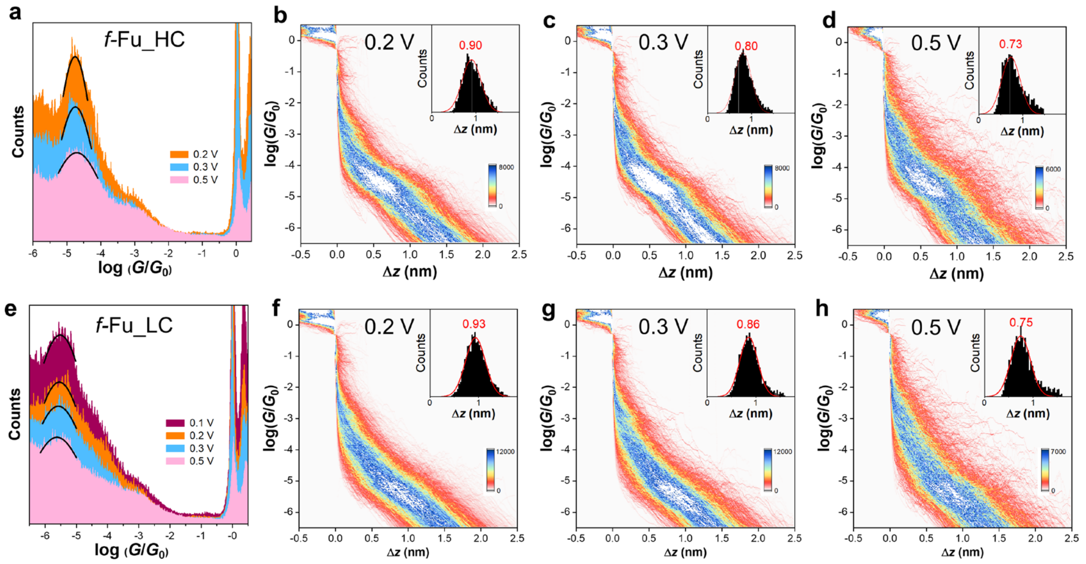

Supplementary Figure 8 Conductance measurement for *f*-Fu under positive biases in THF: TMB (1:4, *v*/*v*). 1D histograms for (**a**) the HC states of *f*-Fu under different biases. 2D histograms for the HC states of *f*-Fu under (**b**) 0.2, (**c**) 0.3 and (**d**) 0.5 V. 1D histograms for (**e**) the LC states of *f*-Fu under different biases. 2D histograms for the LC states of *f*-Fu under (**f**) 0.2, (**g**) 0.3 and (**h**) 0.5 V.

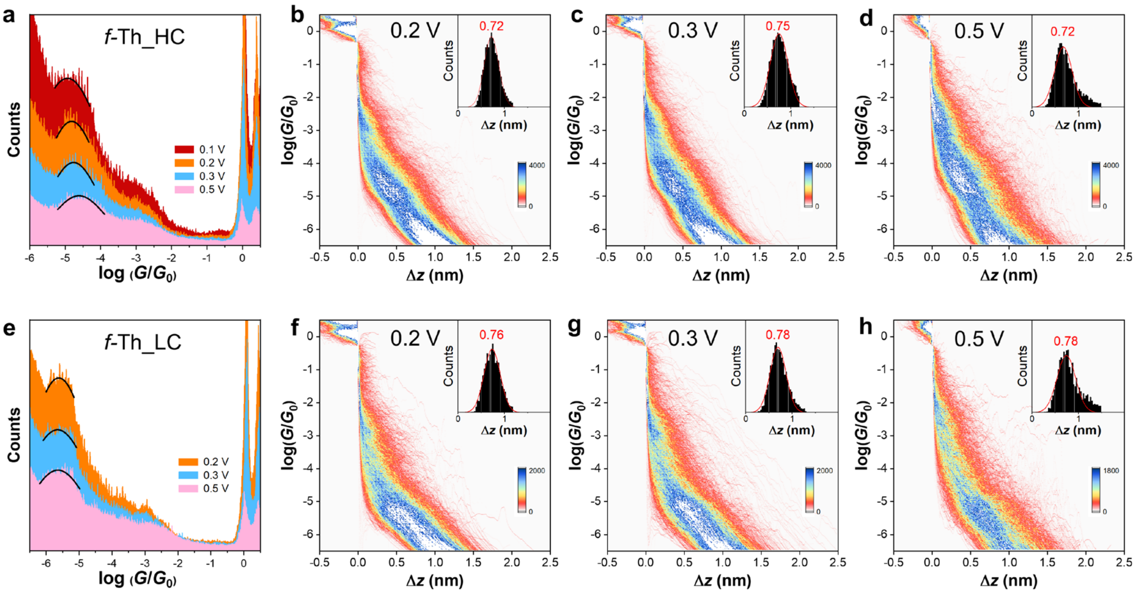

Supplementary Figure 9 Conductance measurement for *f*-Th under positive biases in THF: TMB (1:4, *v*/*v*). 1D histograms for (**a**) the HC states of *f*-Th under different biases. 2D histograms for the HC states of *f*-Th under (**b**) 0.2, (**c**) 0.3 and (**d**) 0.5 V. 1D histograms for (**e**) the LC states of *f*-Th under different biases. 2D histograms for the LC states of *f*-Th under (**f**) 0.2, (**g**) 0.3 and (**h**) 0.5 V.

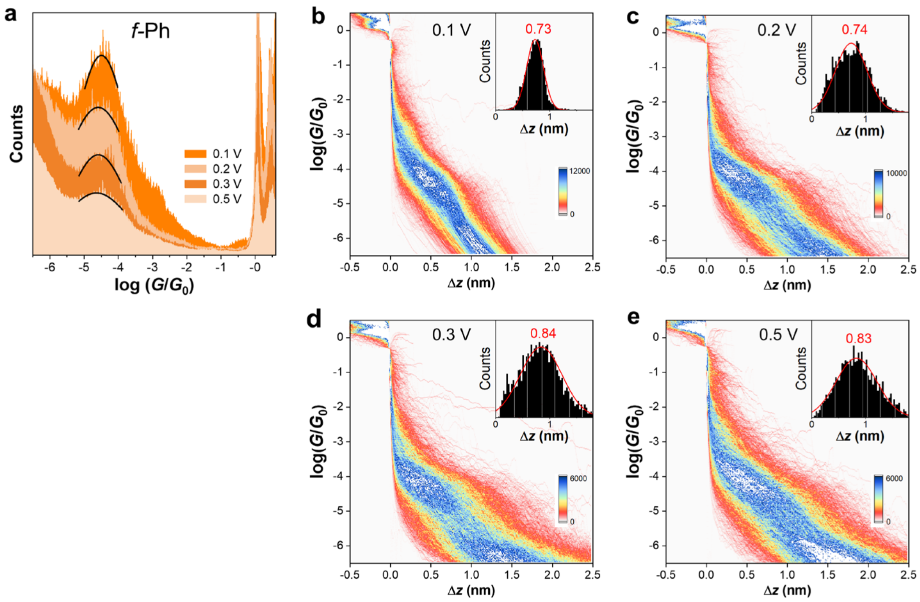

Supplementary Figure 10 Conductance measurement for *f*-Ph under different biases in THF: TMB (1:4, *v*/*v*). 1D histograms for (**a**) *f*-Ph under different biases. 2D histograms for *f*-Ph under (**b**) 0.1, (**c**) 0.2, (**d**) 0.3 and (**e**) 0.5 V.

Supplementary Note 2.2 Conductance measurement for *f*-Fu and *f*-Th under negative biases.

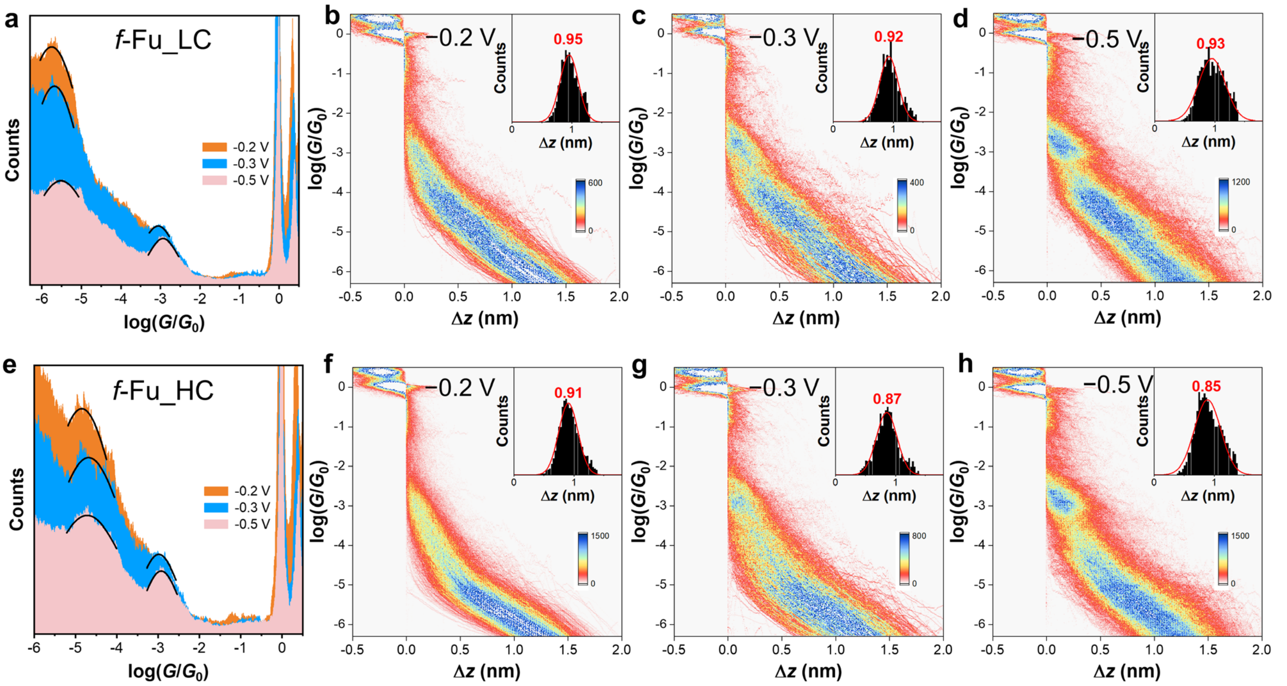

Supplementary Figure 11 Conductance measurement for *f*-Fu under negative biases in THF: TMB (1:4, *v*/*v*). 1D histograms for (**a**) the HC states of *f*-Fu under negative biases. 2D histograms for the HC states of *f*-Fu under (**b**) −0.2, (**c**) −0.3 and (**d**) −0.5 V. 1D histograms for (**e**) the LC states of *f*-Fu under different biases. 2D histograms for the LC states of *f*-Fu under (**f**) −0.2, (**g**) −0.3 and (**h**) −0.5 V.

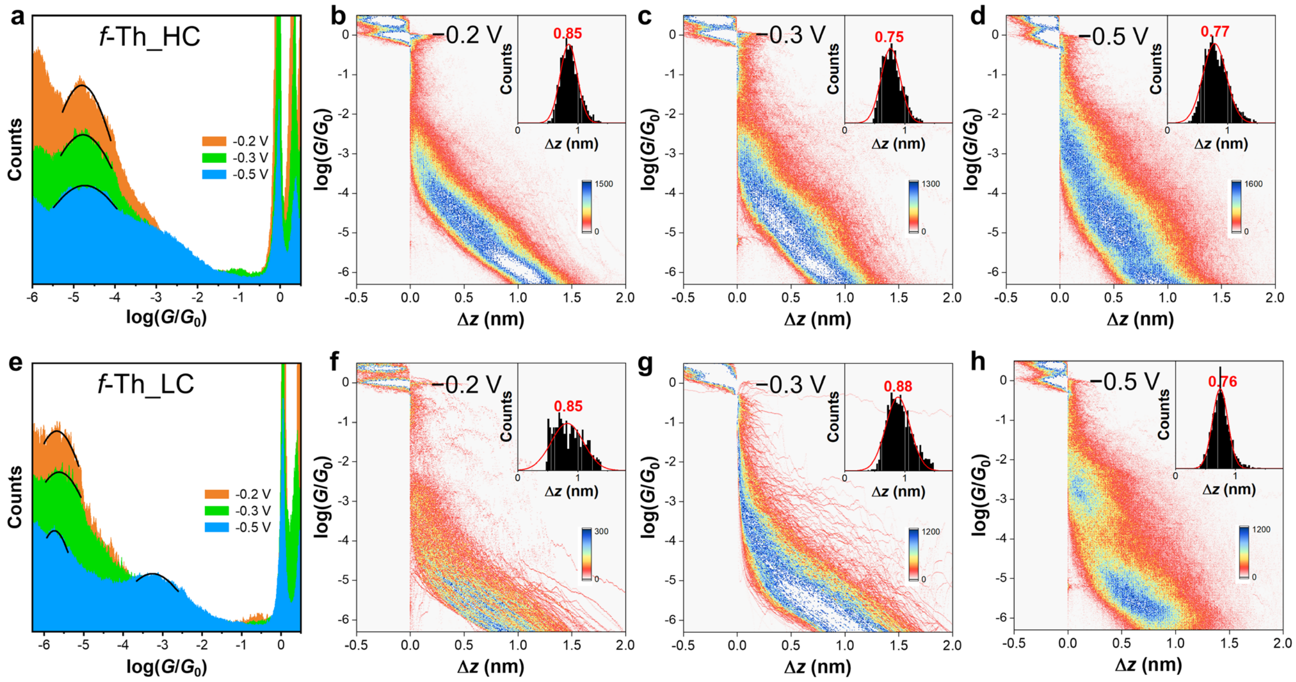

Supplementary Figure 12 Conductance measurement for *f*-Th under negative biases in THF: TMB (1:4, *v*/*v*). 1D histograms for (**a**) the HC states of *f*-Th under negative biases. 2D histograms for the HC states of *f*-Th under (**b**) −0.2, (**c**) −0.3 and (**d**) −0.5 V. 1D histograms for (**e**) the LC states of *f*-Th under different biases. 2D histograms for the LC states of *f*-Th under (**f**) −0.2, (**g**) −0.3 and (**h**) −0.5 V.

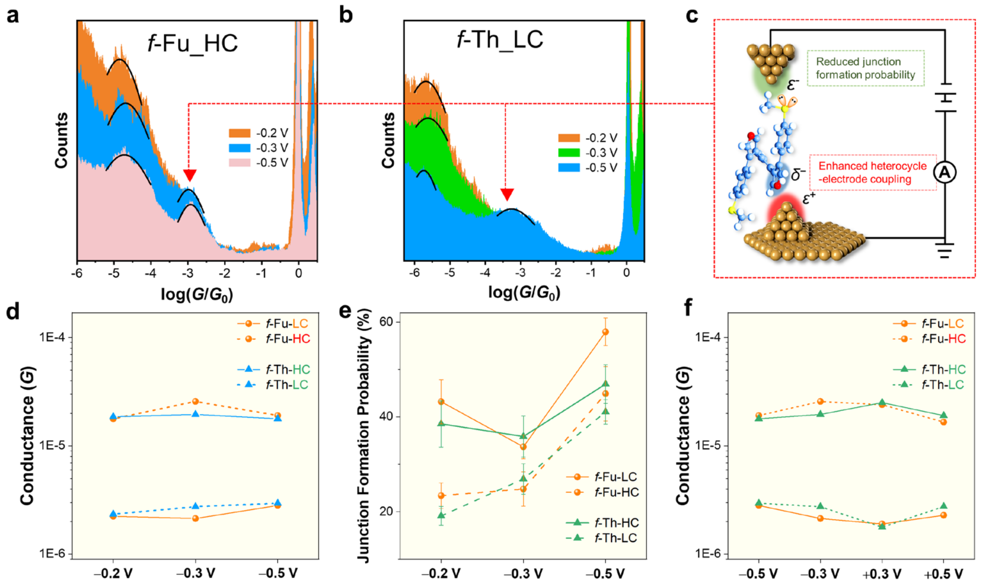

Supplementary Figure 13 Schematic illustration and statistic data of conductance measurement under negative biases for *f*-Fu and *f*-Th in THF: TMB (1:4, *v*/*v*). 1D histograms for (**a**) the HC states of *f*-Fu and (**b**) the LC states of *f*-Th under negative biases. (**c**) Schematic illustration of the probable molecular junction configuration under negative biases corresponding to the additional conductance around 10^{-3} G_0. Statistical data of the conductance (**d**) and junction formation probabilities (**e**) for *f*-Fu and *f*-Th under negative biases. (**f**) Statistical data of the conductance for *f*-Fu and *f*-Th under different applied biases. The error bars are the standard deviation of multiple results for junction formation probabilities in conductance measurment over three times.

Although the switching of conductance is still realizable under intensified negative biases, there are two major differences for the conductance results. The first one is that conductance peak around 10^{-3} G_0 become more distinct under intensified negative biases. Based on the short plateau lengths for 10^{-3} G_0, it is believed that this peak stems from the anchoring of heterocycle and SMe on one stacking arms to the electrodes. Initially, the molecules are scattered on the substrate, and then the tip “picks up” the molecule to form conducting junction. Once the tip is biased negatively, the electrostatic potential of the substrate is positive. Therefore, the electron-rich heterocycle will easily participate in charge transport and their coupling with substrate become stronger too. Hence, during the pick-up procedure of the tip, it becomes much easier to form electron transmission between heterocycles and substrate, which accounts for the distinct peak at 10^{-3} G_0. The peak being more distinguishable at the same voltage condition in the negatively-biased measurement for *f*-Fu also supports this assumption, as the electronegativity of furan is stronger. Therefore, it is easier for furan to couple with the substrate. The second one is that the junction formation probabilities are much lower than those in experiment under positive biases, which can be explained by the enhanced resistance between molecules and the tip. Since the tip is biased negatively, the electrostatic potential of the tip is negative. In the meantime, sulfur is favorable as the anchor in molecular junction because of the existence of lone-pair electron orbital. The electron repulsion between the tip and sulfur is enhanced at negative biases, resulting in lower junction formation probabilities.

Supplementary Note 2.3 Conductance measurement under variable piezo rates for mechanical analysis.

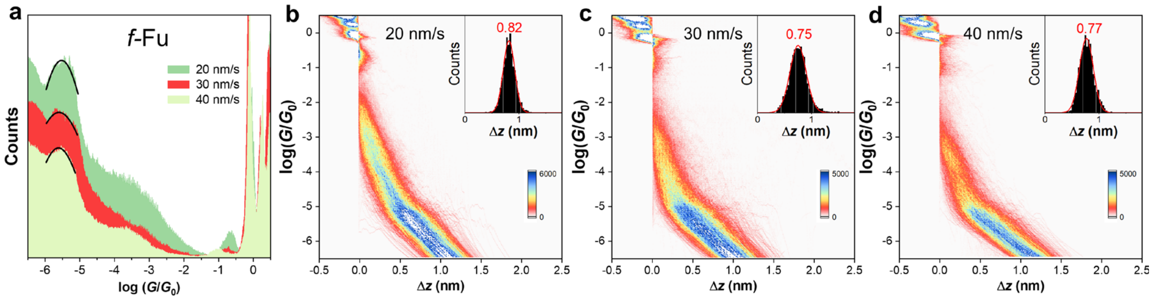

Supplementary Figure 14 Conductance measurement with different piezo rates. (**a**) 1D histograms of conductance measurement in THF: TMB (1:4, *v*/*v*) under different piezo rates for *f*-Fu. 2D histograms of conductance measurement for *f*-Fu under piezo rates of (**b**) 20, (**c**) 30 and (**d**) 40 nm s^{-1}, respectively. Insets are the relative displacement distribution histograms with Gaussian fittings.

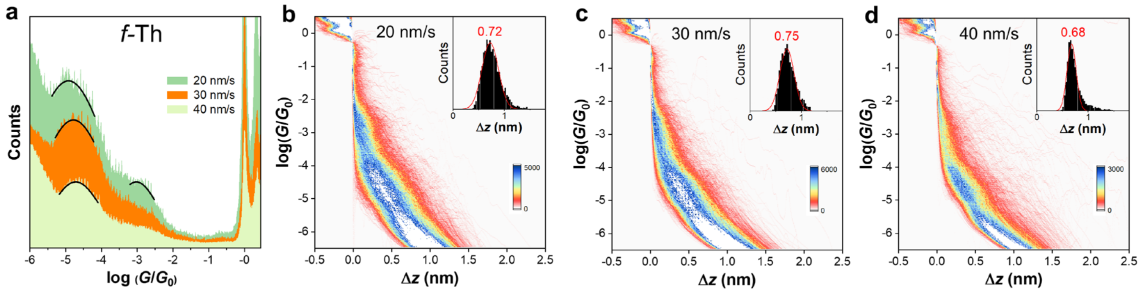

Supplementary Figure 15 Conductance measurement with different piezo rates. (**a**) 1D histograms of conductance measurement in THF: TMB (1:4, *v*/*v*) under different piezo rates for *f*-Th. 2D histograms of conductance measurement for *f*-Fu under piezo rates of (**b**) 20, (**c**) 30 and (**d**) 40 nm s^{-1}, respectively. Insets are the relative displacement distribution histograms with Gaussian fittings.

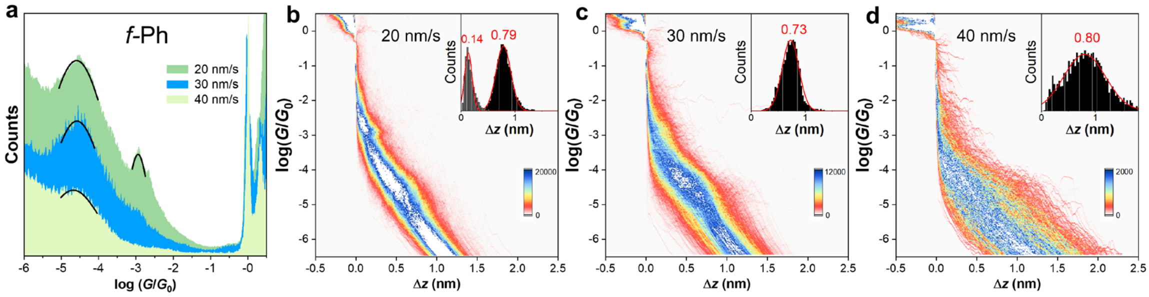

Supplementary Figure 16 Conductance measurement with different piezo rates. (**a**) 1D histograms of conductance measurement in THF: TMB (1:4, *v*/*v*) under different piezo rates for *f*-Ph. 2D histograms of conductance measurement for *f*-Fu under piezo rates of (**b**) 20, (**c**) 30 and (**d**) 40 nm s^{-1}, respectively. Insets are the relative displacement distribution histograms with Gaussian fittings.

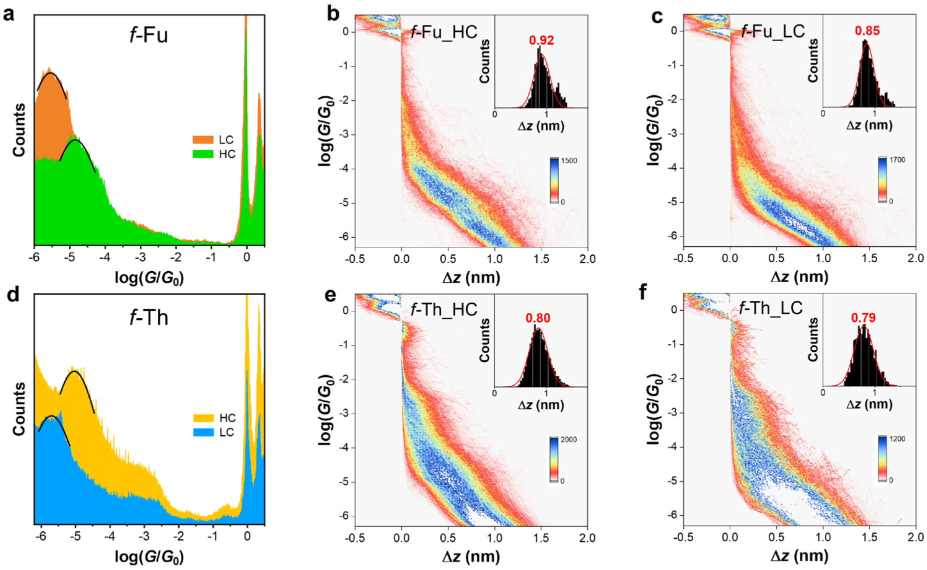

Supplementary Figure 17 Conductance measurement at 0.2 V with the piezo rate of 40 nm s^{-1}. 1D histograms of HC states and LC states for *f*-Fu (**a**) in THF: TMB (1:4, *v*/*v*). 2D histograms of HC states (**b**) and LC states (**c**) for *f*-Fu. 1D histograms of HC states and LC states for *f*-Th (**d**) in THF: TMB (1:4, *v*/*v*). 2D histograms of HC states (**e**) and LC states (**f**) for *f*-Th.

Considering the piezo rates has a profound effect on the junction formation probability, the piezo rates applied in STM-BJ measurement need to be selected discreetly. The piezo rates of 20, 30 and 40 nm s^{-1} are applied in conductance measurement of *f*-Fu, *f*-Th and *f*-Ph in tetrahydrofuran/mesitylene (THF/TMB, 1:4, *v*/*v*). Only one-distinct conductance peak can be found under different piezo rates in the measurement of *f*-Fu (Supplementary Fig. 11a), but an ambiguous shoulder around $10^{-3.5}$ G_0 appears under 20 nm s^{-1}, assuming that the oxygen (O) atom may attach to electrodes under slow rate and it is the conductance of one arm in *f*-Fu. Since Au-O coordination is quite weak, this shoulder may vanish under higher piezo rates. Under a piezo rate of 20 nm s^{-1}, *f*-Ph exhibits multiple conductance, consistent with the previous observation[1] because of the presence of multiple conformations (Supplementary Fig. 11a). And the junction formation probability is decreased under 40 nm s^{-1}. According to the discussion above, the piezo rate is set at 30 nm s^{-1}, applied in all other measurements.

In addition to verify that the switching of conducting states does not originate from the mechanical force, conductance measurements at 0.2 V under a piezo rate of 40 nm s^{-1} are also performed. Both HC states and LC states for *f*-Fu and *f*-Th are still observable. When lowering the piezo rate at 0.1 V, the switching does not happen but just one conductance peak is detected for these foldamers. These results comprehensively illustrate that the switching is induced by voltage rather than mechanical force.

Supplementary Note 2.4 Further discussion on control molecules

The attribution of conducting states for *f*-Th-H

f-Th-H exhibits two blurry conductance peaks at $10^{-2.97}$ and $10^{-5.07}$ G_0, with junction lengths of 0.56 and 0.108 nm after calibration, respectively. To decipher the origin of these states, an extra linear molecule (*l*-Th) is applied for conductance measurement for comparison. *l*-Th was reported previously[4] to have one relatively higher conductance with shorter junction length referring to the conductance of the monomer and the other relatively lower conductance with longer junction length referring to the conductance of the dimer. In this measurement, *l*-Th also exhibits two conducting states at $10^{-2.72}$ and $10^{-4.66}$ G_0 with junction lengths of 0.72 and 1.17 nm, respectively, which is similar to

previous report[4] (Supplementary Fig. 15c and 15d). Therefore, the conductance at $10^{-2.72}$ G_0 belongs to the monomer *l*-Th corresponding to one arm of *f*-Th-H while the conductance at $10^{-4.66}$ G_0 belongs to the dimer configuration, which is similar the folded configuration of *f*-Th-H.

In that case, it is reasonable to presume the conductance state at $10^{-2.97}$ G_0 stems from the contacting configuration that the thiomethyl group (SMe) attaches to one electrode and the thiophene is connected to the other. Considering the stacking arms provide certain steric hindrance and the distance between SMe and the sulfur atom in thiophene in single arm is slightly longer than 0.56 nm (around 0.8 nm), the thiophene probably attaches to the electrode as an ensemble aromatic ring. It also can explain the slightly reduced conductance of *f*-Th-H in comparison with that of *l*-Th and the blurry conductance peaks. As for the peak at $10^{-5.07}$ G_0, it may come from the through-space charge injection *via* terminal benzene (Supplementary Fig. 15b) in the consideration of the similarity to the conductance of *l*-Th dimer. The absence of one SMe is responsible for the weakened conductivity and shorter statistic junction length of *f*-Th-H. The other possibility for $10^{-5.07}$ G_0 is that the molecule unfolds, and thus the thiophene gets far from SMe group attaching to the electrode. But this explanation is excluded because the transmission along through-bond pathway usually exhibits a flatter conductance density cloud in 2D histogram and a steadier molecular junction.

In conclusion, two blurry conductance peaks around 10^{-3} and 10^{-5} G_0 are attributed to the anchoring of SMe and thiophene to the electrodes and through-space charge injection *via* terminal benzene, respectively.

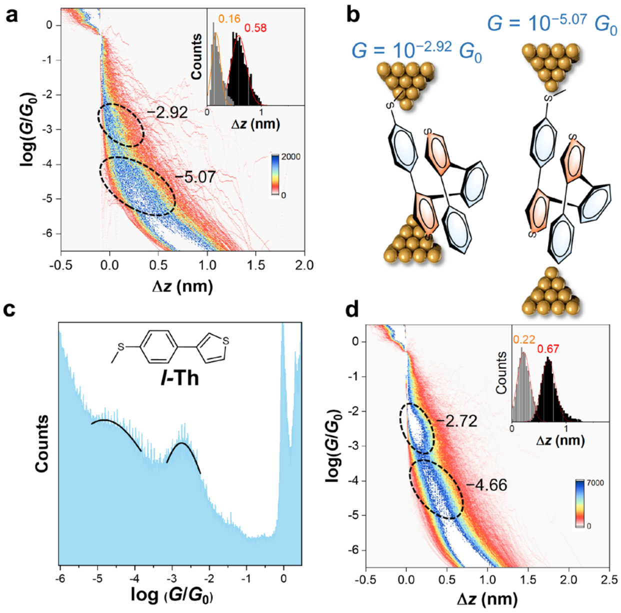

Supplementary Figure 18 Conductance measurements of *f*-Th-H and *l*-Th. (**a**) 2D histograms of conductance measurement for *f*-Th-H. (**b**) Schematic illustration of contacting junction configuration corresponding to two different conducting states for *f*-Th-H. (**c**) 1D histograms of conductance measurement in THF: TMB (1:4, *v*/*v*) for *l*-Th. (**d**) 2D histograms of conductance measurement for *l*-Th.

The attribution of conducting states for *Z*-Th

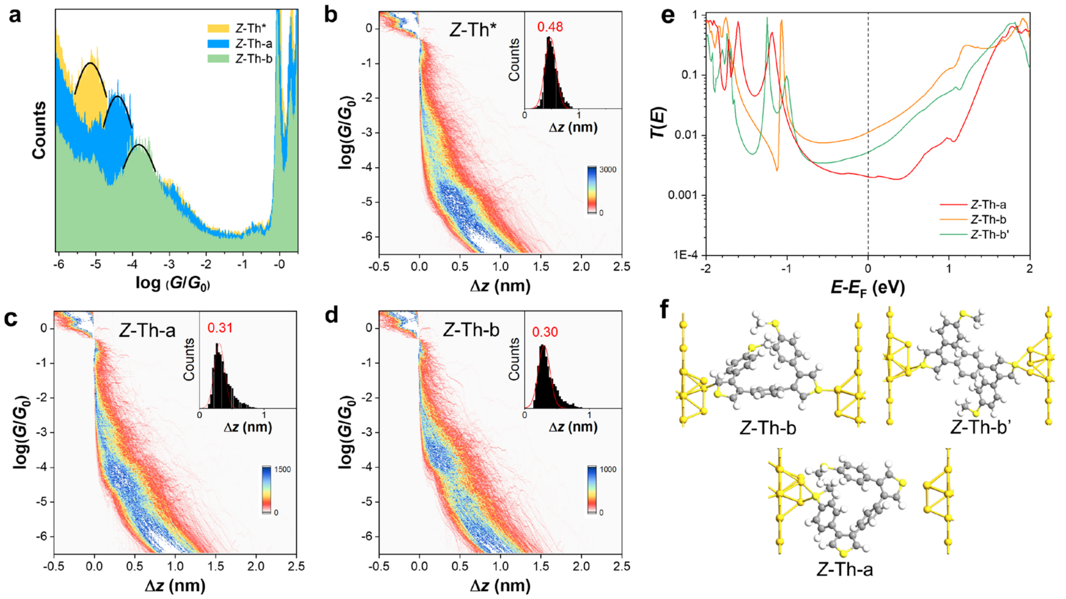

Supplementary Figure 19 Conductance measurement for *Z*-Th. (**a**) 1D histograms of conductance measurement in THF: TMB (1:4, *v*/*v*) for *Z*-Th. 2D histograms of conductance measurement for contacting geometry of (**b**) *Z*-Th*, (**c**) *Z*-Th-a and (**d**) *Z*-Th-b, respectively. *Z*-Th* refers to the contacting geometry that two SMe anchors attach to the electrode. (**e**) Transmission function for *Z*-Th with different contacting geometries. Insets are the relative displacement distribution histograms with Gaussian fittings. (**f**) The contacting geometry for *f*-Th that is not anchored by the two SMe groups.

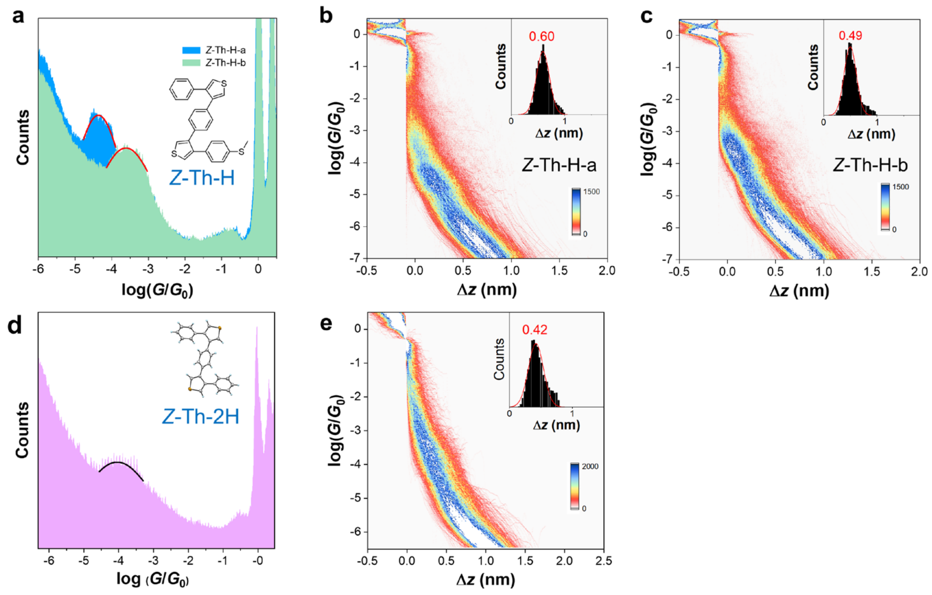

Supplementary Figure 20 Conductance measurement for *Z*-Th-H and *Z*-Th-2H. (**a**) 1D histograms of conductance measurement in THF: TMB (1:4, *v*/*v*) for *Z*-Th-H. 2D histograms of conductance measurement for *Z*-Th-H-a (**b**) and *Z*-Th-H-b (**c**). Insets are the relative displacement distribution histograms with Gaussian fittings. (**d**) 1D histograms of conductance measurement in THF: TMB (1:4, *v*/*v*) for *Z*-Th-2H. (**e**) 2D histograms of conductance measurement for *Z*-Th-2H.

There are three conducting states can be found during the STM-BJ measurement for *Z*-Th, which are $10^{-3.72}$, $10^{-4.15}$ and $10^{-5.00}$ G_0, respectively. Since the conductance of *l*-Th is $10^{-2.72}$ G_0, those three kinds of results are not likely to correspond to the contacting geometry that one arm of *Z*-Th attaches to the electrodes. According to the junction length calculated from plateau lengths and simulated transmission results, it is concluded the conductance of $10^{-3.72}$ originates from the junction with two thiophenes anchored to electrodes as shown in Supplementary Fig. 16f. The linear and planar structure is indicative of better conjugation of *Z*-Th. The similar conductance result of *Z*-Th-2H at $10^{-3.82}$ G_0 further verifies the conclusion above.

The absence of 10^{-5} G_0 in the measurement of *Z*-Th-H reveals that the conductance of 10^{-5} G_0 originates from the junction with two SMe anchored to electrodes, which is marked as "*Z*-Th*" for comparison with foldamers in control experiment. Excluding all other possibility, it is believed that $10^{-4.15}$ G_0 corresponds to the conductance stemming from the junction with one SMe and one thiophene anchored to electrodes, and the referring thiophene is far away from SMe group rather than directly covalently bonded with thiomethyl benzene. The contacting geometry is shown in Supplementary Fig. 16f. In addition, all of these three states remain unchanged under variable biases.

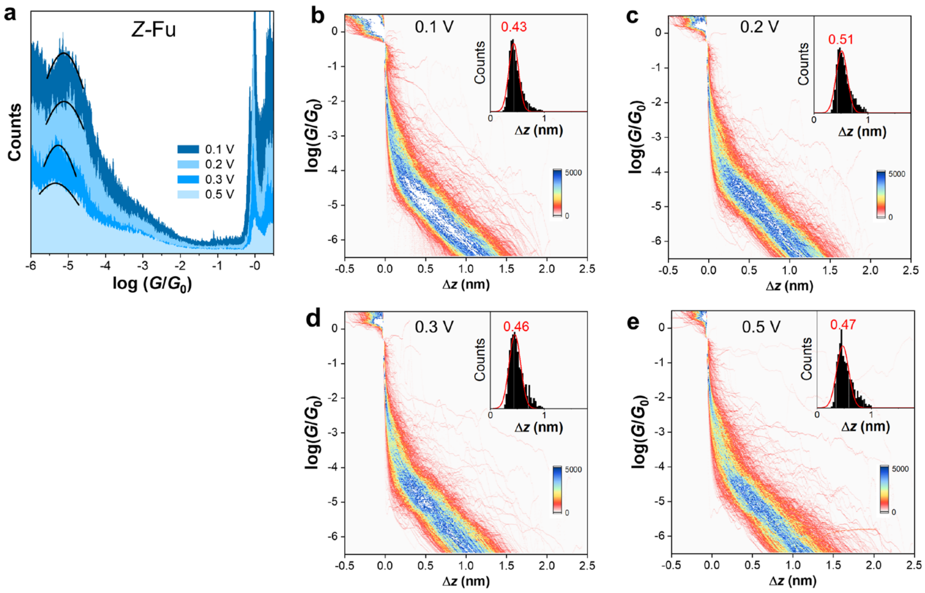

Supplementary Figure 21 Conductance measurement for *Z*-Fu under different biases in THF: TMB (1:4, *v*/*v*). 1D histograms for (**a**) *Z*-Fu under different biases. 2D histograms for *Z*-Fu under (**b**) 0.1, (**c**) 0.2, (**d**) 0.3 and (**e**) 0.5 V.

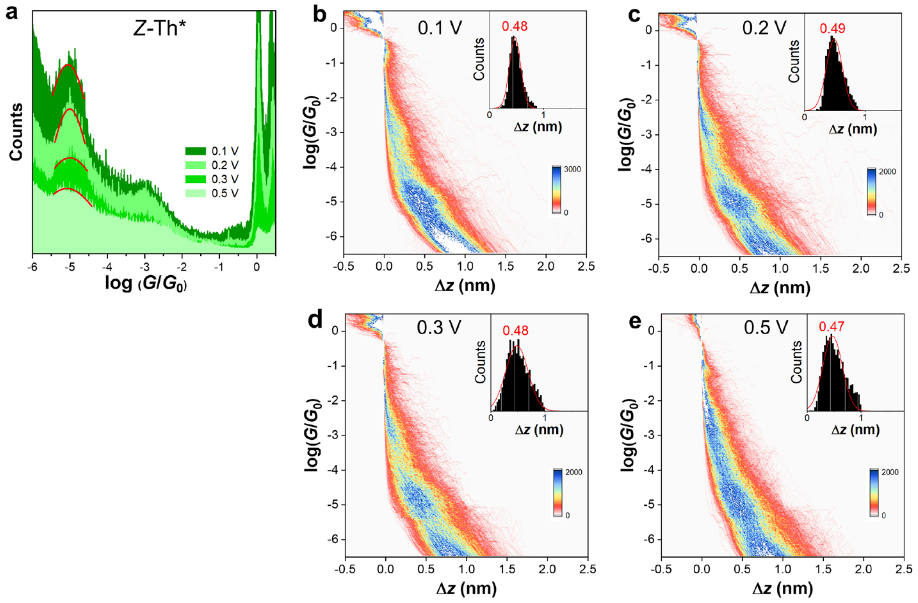

Supplementary Figure 22 Conductance measurement for *Z*-Th* corresponding to the contacting geometry that two SMe anchors attach to the electrodes under different biases in THF: TMB (1:4, *v*/*v*). 1D histograms for (**a**) *Z*-Th under different biases. 2D histograms for *Z*-Th under (**b**) 0.1, (**c**) 0.2, (**d**) 0.3 and (**e**) 0.5 V.

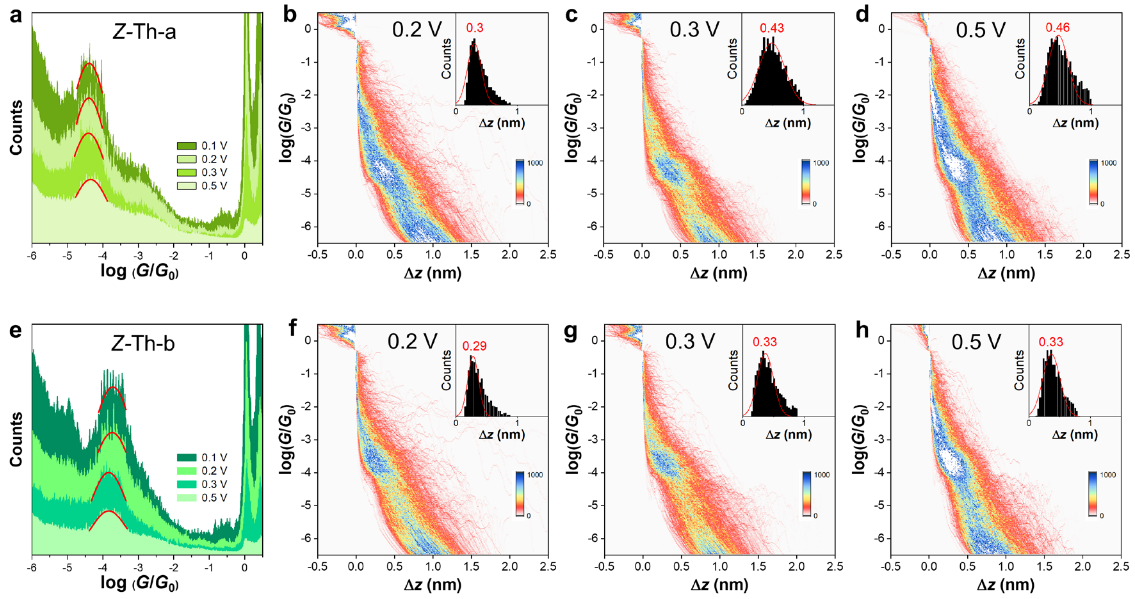

Supplementary Figure 23 Conductance measurement for *Z*-Th under different biases in THF: TMB (1:4, *v*/*v*). 1D histograms for (**a**) *Z*-Th-a under different biases. 2D histograms for *Z*-Th-a under (**b**) 0.2, (**c**) 0.3, (**d**) 0.5 V. 1D histograms for (**e**) *Z*-Th-b under different biases. 2D histograms for *Z*-Th-b under (**f**) 0.2, (**g**) 0.3, (**h**) 0.5 V.

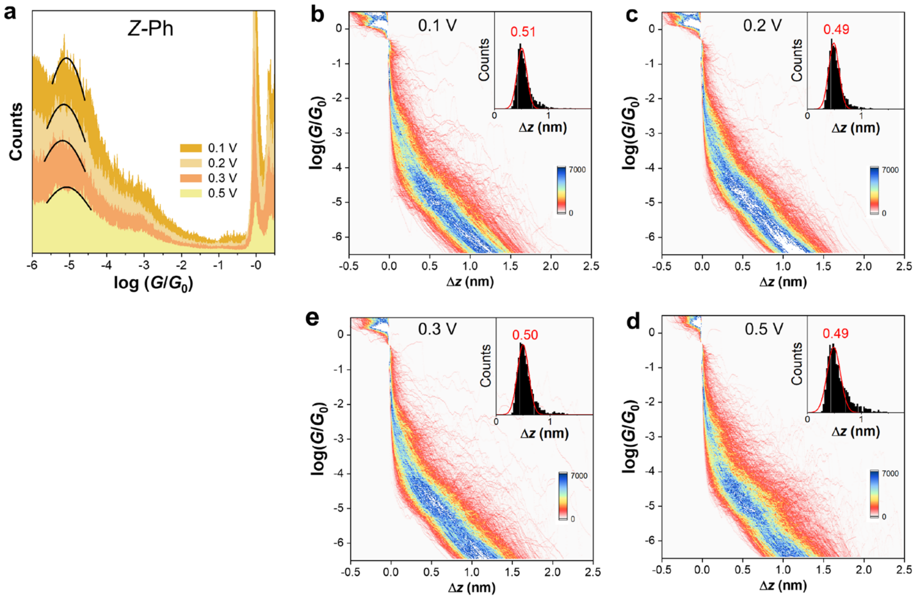

Supplementary Figure 24 Conductance measurement for *Z*-Ph under different biases in THF: TMB (1:4, *v*/*v*). 1D histograms for (**a**) *Z*-Ph under different biases. 2D histograms for *Z*-Ph under (**b**) 0.1, (**c**) 0.2, (**d**) 0.3 and (**e**) 0.5 V.

Supplementary Note 2.5 Further discussion of the existence of intermolecular through-space interaction during junction formation

This work primarily focuses on intramolecular through-space conjugation, and thus excluding the intermolecular through-space interaction is quite significant. On one hand, even in relatively closer packing for single crystal than in solution (Supplementary Fig. 22), there is no intermolecular π-stacking in crystal cell as the closest distances between inter-aromatic rings are longer than 3.5 Å and just a few negligible C-H···π interaction. The folded and twisted structure is helpful to inhibit intermolecular π-stacking. Unlike the planar *l*-Th with a very small torsion angle of 3.72° between thiophene and benzene, the stacked arms in *f*-Fu and *f*-Th hold torsion angles larger than 20°, which are not favorable for the formation of intermolecular through-space interaction.

Additional conductance measurements are also designed to gain in-depth insights. The intermolecular π-stacking usually depends on the molecular concentration, and the probability of stacking increases with the enhanced concentration. But in conductance experiments with a higher concentration (1 mM), it is found that both heterocycle-benzene stacking foldamers remain single conductance peak at 0.1 V as well as co-existence of dual-states at 0.3 V (Supplementary Fig. S23 and S24), and the HC state for *f*-Fu and LC state for *f*-Th stay as minor states, respectively. The only difference is that the peak at 10^{-3} G_0 becomes more distinct in the experiment with higher concentrations, but this peak is too short to be the conductance of dimers' junction.

What's more, the measurements of *l*-Fu and *l*-Th that are believed to form dimers' junction are also performed under different biases. Previous reports certify that these linear conjugated molecules are capable of forming dimers and transporting charges effectively under intensified external electric field. The dimers' junction is much longer that the junction length of monomer with length difference over 3 Å. For example, the experimental difference in junction length between monomer and dimer of *l*-Th is 4.5 Å. However, in the measurement of *f*-Fu and *f*-Th, the differences between two conducting states usually less than 1 Å, which is unlikely to be the difference between monomer and dimer.

In this regard, all kinds of conducting states do not belong to the dimer or trimer that form intermolecular

interaction. There is no observable intermolecular through-space interaction in foldamer' experiment.

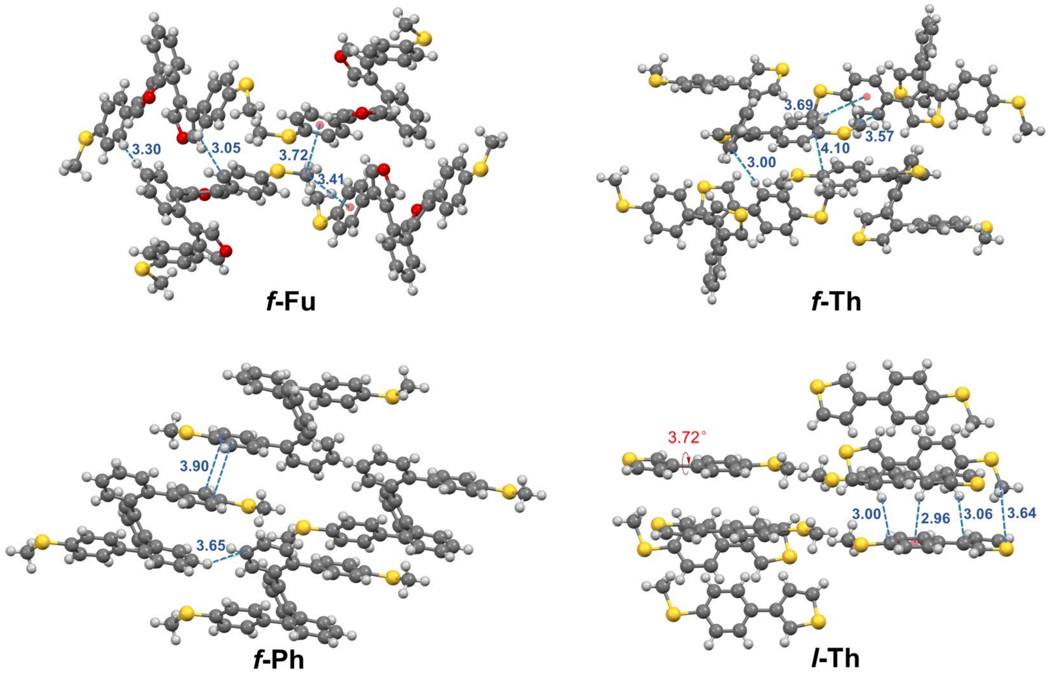

Supplementary Figure 25 The investigations on intermolecular through-space interaction of foldamers and linear *l*-Th. The crystal packing of *f*-Fu and *f*-Th, *f*-Ph and *l*-Th with labelled atomic distances and torsion angles.

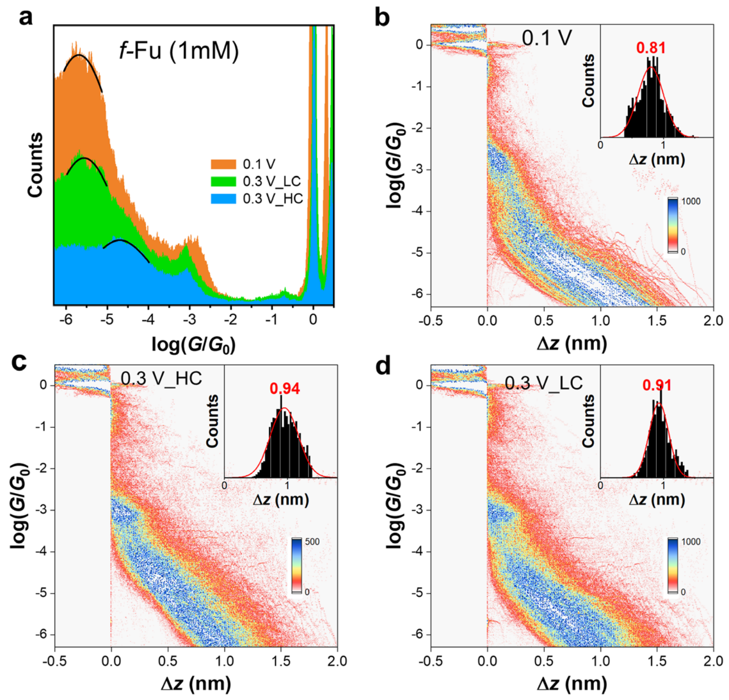

Supplementary Figure 26 Conductance measurement for *f*-Fu with the concentration of 1 mM in THF: TMB (1:4, *v*/*v*). 1D histograms for (**a**) *f*-Fu under different biases. 2D histograms for LC states of *f*-Fu under (**b**) 0.1 and (**c**) 0.3 V, and HC states for *f*-Fu under 0.3 V (**d**).

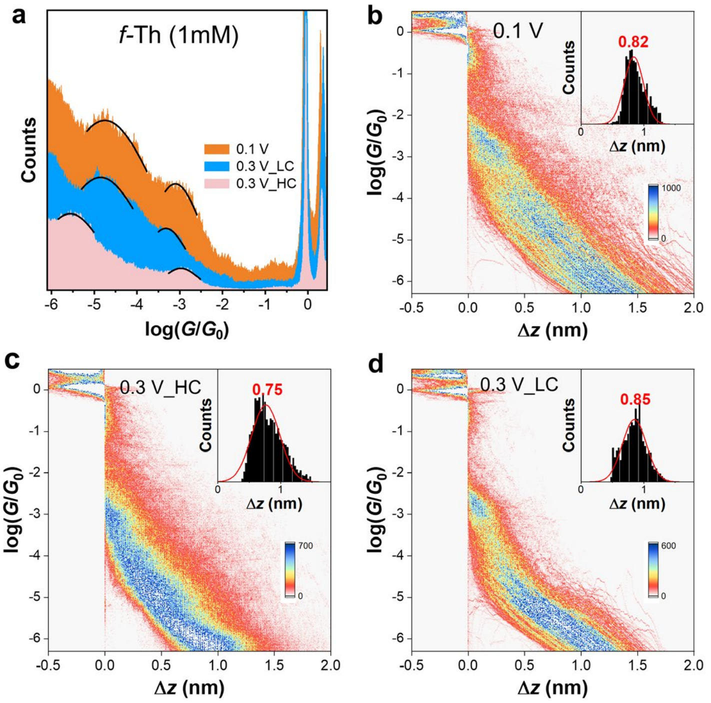

Supplementary Figure 27 Conductance measurement for *f*-Th with the concentration of 1 mM in THF: TMB (1:4, *v*/*v*). 1D histograms for (**a**) *f*-Fu under different biases. 2D histograms for LC states of *f*-Fu under (**b**) 0.1 and (**c**) 0.3 V, and HC states for *f*-Fu under 0.3 V (**d**).

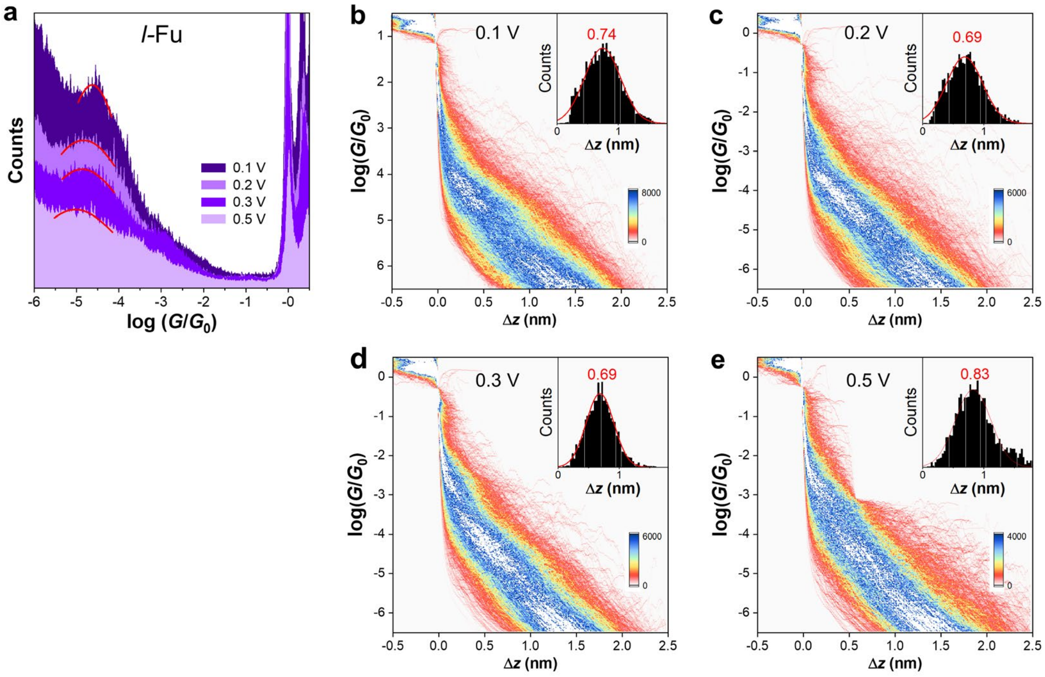

Supplementary Figure 28 Conductance measurement for *l*-Fu under different biases in THF: TMB (1:4, *v*/*v*). 1D histograms for (**a**) *l*-Fu under different biases. 2D histograms for *l*-Fu under (**b**) 0.1, (**c**) 0.2, (**d**) 0.3 and (**e**) 0.5 V.

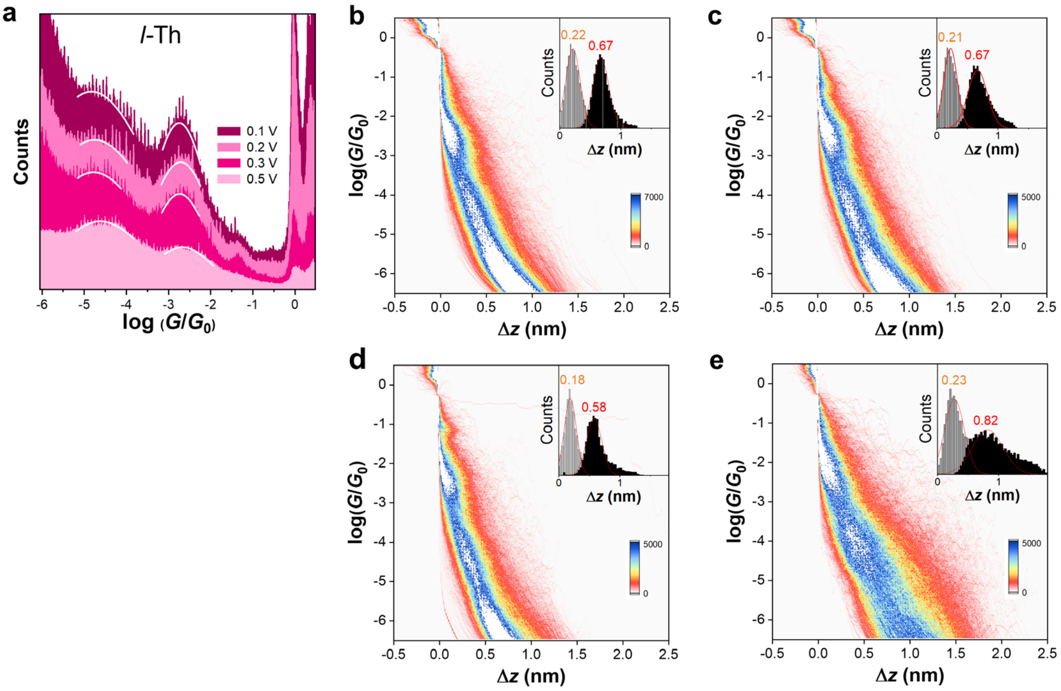

Supplementary Figure 29 Conductance measurement for *l*-Th under different biases in THF: TMB (1:4, *v*/*v*). 1D histograms for (**a**) *l*-Th under different biases. 2D histograms for *l*-Th under (**b**) 0.1, (**c**) 0.2, (**d**) 0.3 and (**e**) 0.5 V.

Supplementary Note 3. Theoretical calculation

Molecular optimization, Gibbs free energy calculation and electrostatic potential distribution

The molecular optimization was carried out by Gaussian16 package using M06-2x/6-311G(d, p) basis set with DFT-D3 correction. And the Gibbs free energy was calculated using M06-2x/def2TZVP with DFT-D3 correction based on the optimized folded or unfolded conformation. The electrostatic potential distribution was calculated by Multifwn. after molecular optimization under electric field with different intensity. The direction of external electric field was set along the alignment of two sulfur atoms of SMe groups.

Energy decomposition based molecular force field

The energy decomposition of non-covalent interaction was carried out by Multifwn[5] based on molecular force field. The adopted force field for organic system was AMBER force field. The ensemble energy of non-bonding interaction was divided into the terms of electrostatic interaction, Pauli repulsion and dispersion.

Junction simulation based on ATK software.

In junctions based on conjugated organic molecules operating near room temperature, the current is quantum coherent and elastic. In the linear response regime, where the applied bias is small compared to chemical potential of the electrodes and the temperature is lower than the operating temperature, the differential conductance may be expressed as[6]

$$\frac{dI}{dV} = G_0 \int dE(-\frac{\partial f_0}{\partial E}) Tr\{T(E)T^{\dagger}(E)\} \quad (1)$$

where G_0 (77.6 μS) is the quantum conductance, $f_0(E)$ is the Fermi function of the electrodes at zero bias, $T(E)$ is the transmission amplitude. Within the nonequilibrium Green's function (NEGF) framework, the molecular junction can be decomposed into three parts: the left and right electrodes and the central scattering region. The electronic structures and Green's function of macroscopic electrodes as infinite periodic bulks are computed from Kohn-Sham Density Functional Theory (KS-DFT). And the electrodes are connected to the central scattering region through the self-energy terms.

Since molecular conductance is highly sensitive to contacting configuration of metal-molecule-metal junction, different junctions based on different molecular conformations are simulated and the most probable theoretical junction configurations are figured out, corresponding to experimental results. Given the GGA-PBE exchange-correlation functional tends to underestimated energy gaps between HOMOs and LUMOs, the theoretical results usually overestimate the conductance and Fermi-level pinning effects. However, it has been shown to characterize the chemical trends and transport ratios in the system we consider here. Molecular projection self-consistent Hamilton is also calculated to unveil the electronic structures of organic molecules. And the isosurface is 0.04 if not specified.

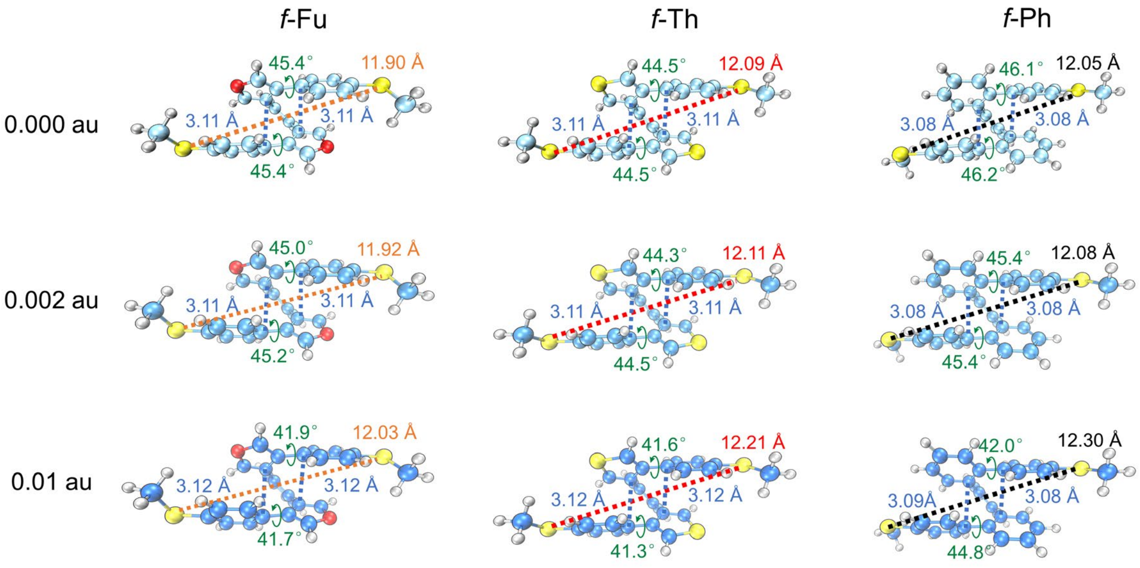

Supplementary Figure 30 The optimized molecular geometries of *f*-Fu, *f*-Th and *f*-Ph in different electric field. The direction of electric field points from the sulfur atom of the left SMe anchors to the sulfur atom of the right SMe anchors.

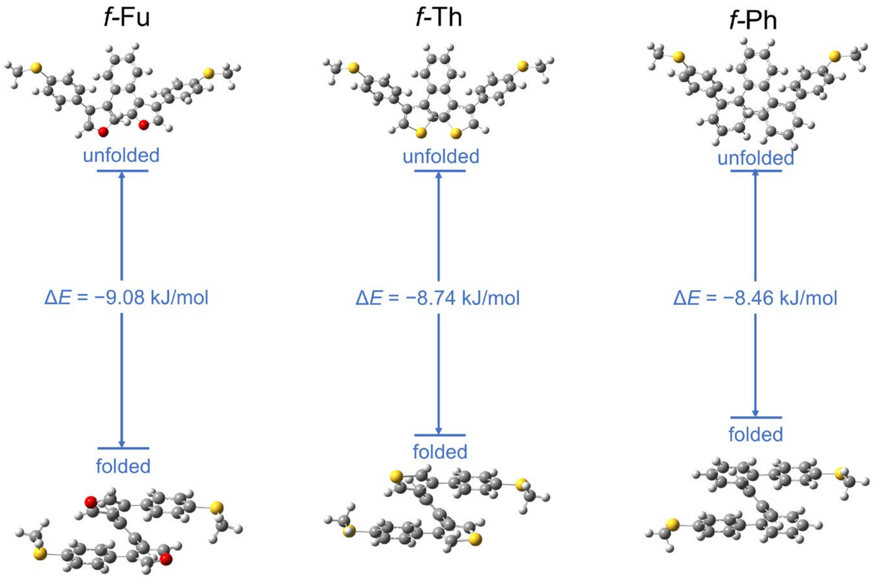

Supplementary Figure 31 The molecular structures and energy differences between unfolded and folded conformations.

Supplementary Table 1. Calculated non-covalently interacted energy of *π*–*π* stacking (Type I) and edge-to-face interaction (Type II).

			E_{elec} (kcal/mol)	E_{Pauli} (kcal/mol)	E_{disp} (kcal/mol)	E_{tot} (kcal/mol)
***f*-Fu**	Type I	0.000 au	6.93	63.42	-85.38	-15.04
		0.002 au (unrelaxed)	7.15	63.42	-85.38	-14.81
		0.002 au (relaxed)	6.18	63.58	-85.55	-15.79
	Type II	0.000 au	-1.16	19.01	-27.42	-9.57
		0.002 au (unrelaxed)	-1.28	19.01	-27.42	-9.68
		0.002 au (relaxed)	-1.16	19.08	-27.42	-9.51
***f*-Th**	Type I	0.000 au	6.25	56.81	-81.82	-18.76
		0.002 au (unrelaxed)	6.08	56.81	-81.82	-18.94
		0.002 au (relaxed)	5.69	56.98	-82.03	-19.35
	Type II	0.000 au	-1.06	26.34	-31.59	-6.30
		0.002 au (unrelaxed)	-1.07	26.34	-31.59	-6.32
		0.002 au (relaxed)	-1.29	26.43	-31.66	-6.50
***f*-Ph**	Type I	0.000 au	8.40	62.30	-88.58	-17.88
		0.002 au (unrelaxed)	8.14	62.30	-88.58	-18.14
		0.002 au (relaxed)	6.77	62.06	-88.11	-19.27
	Type II	0.000 au	-2.48	35.21	-35.93	-3.19
		0.002 au (unrelaxed)	-2.76	35.21	-35.93	-3.47
		0.002 au (relaxed)	-3.14	-36.27	-36.50	-3.37

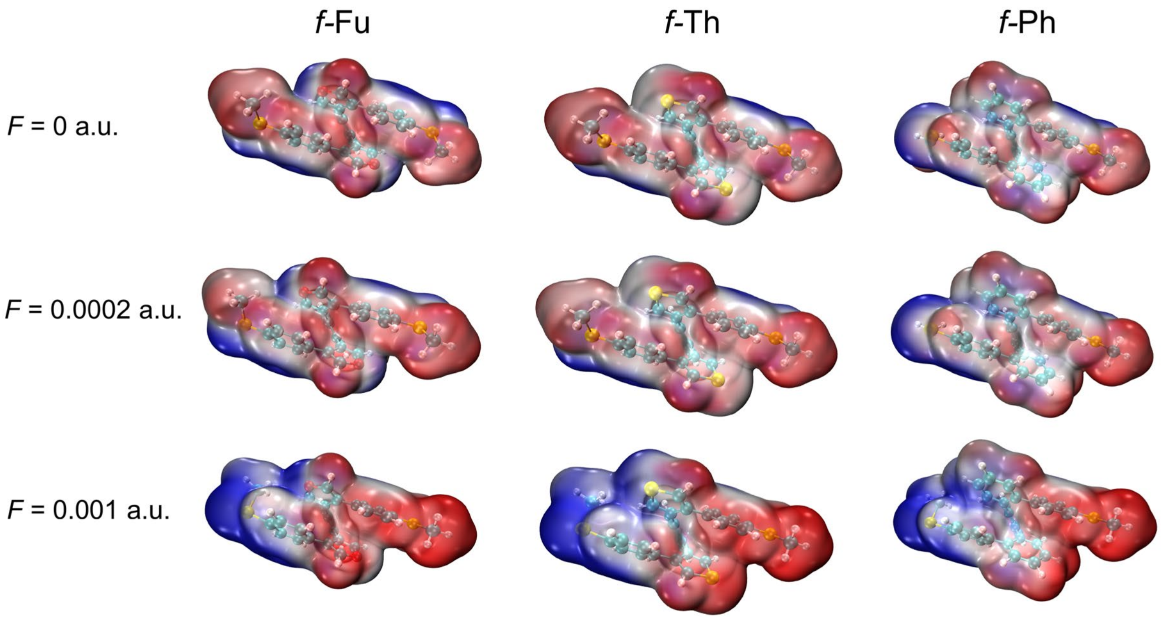

Supplementary Figure 32 The electrostatic potential distribution for *f*-Fu, *f*-Th and *f*-Ph under different intensities of electric field. The direction of electric field points from the sulfur atom of the left SMe anchors to the sulfur atom of the right SMe anchors.

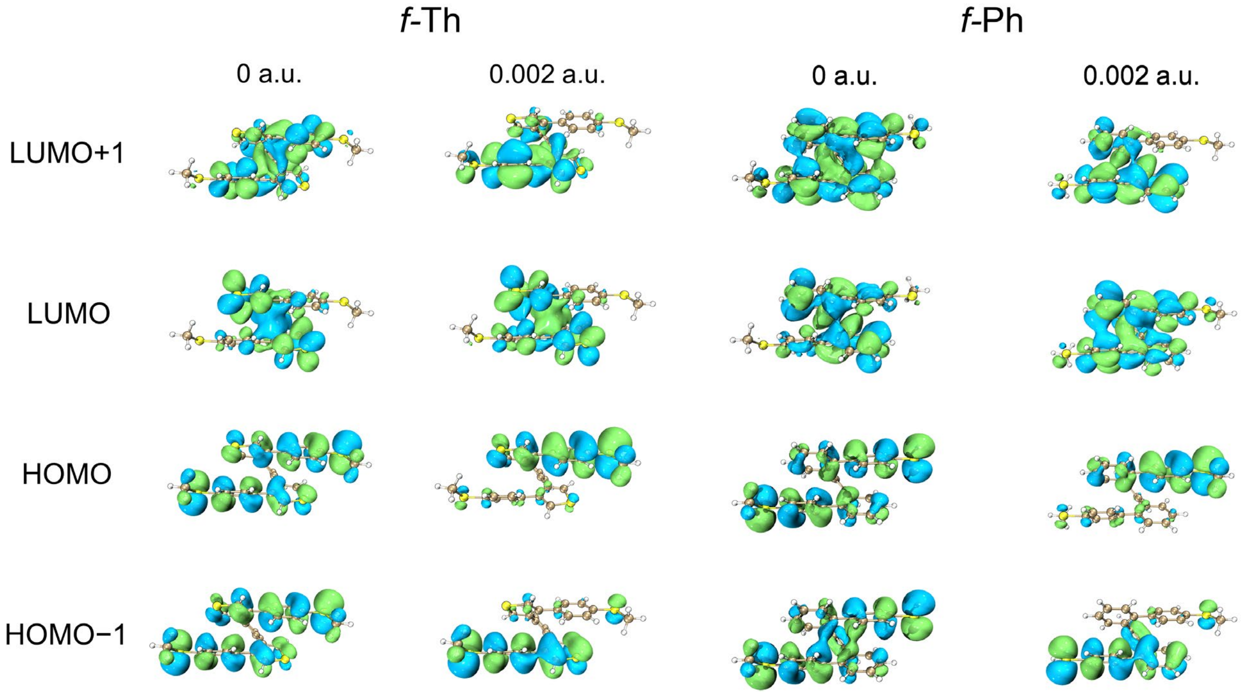

Supplementary Figure 33 Molecular orbitals for *f*-Th and *f*-Ph under different intensities of electric field. The isovalue is 0.02.

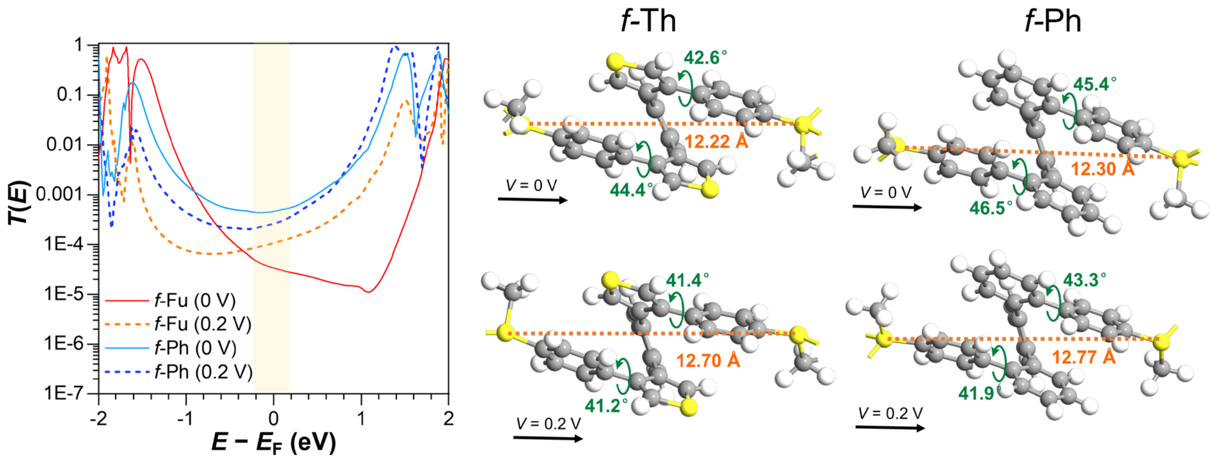

Supplementary Figure 34 The transmission functions and optimized junction geometries for *f*-Ph and *f*-Fu at 0 and 0.2 V, respectively. And the optimized junction geometries for *f*-Th and *f*-Ph at 0 and 0.2 V.

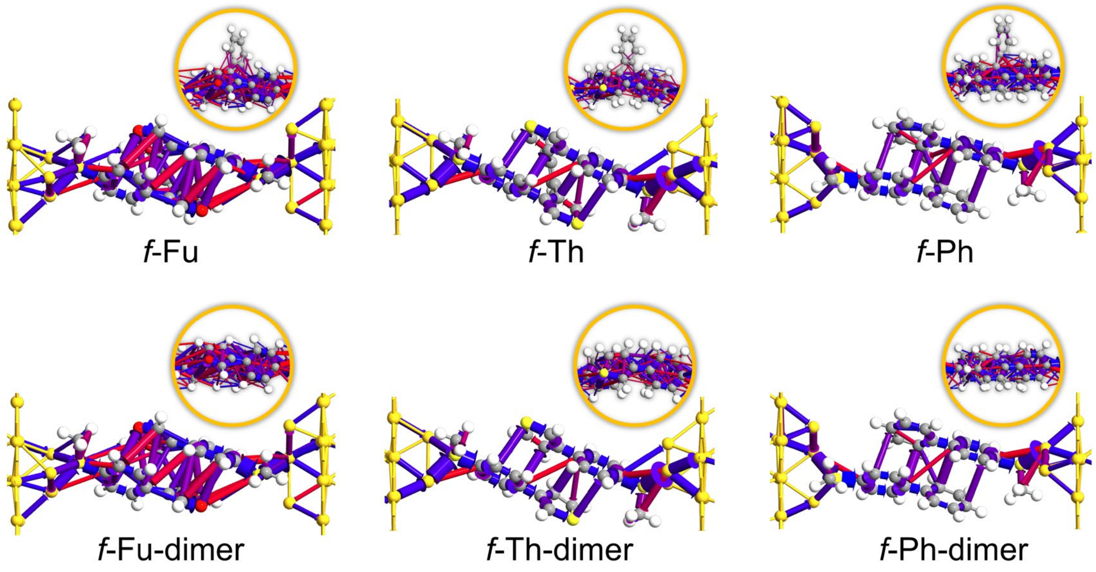

Supplementary Figure 35 The transmission pathway analyses of *f*-Fu, *f*-Th, *f*-Ph, *f*-Fu-dimer, *f*-Th-dimer and *f*-Ph-dimer.

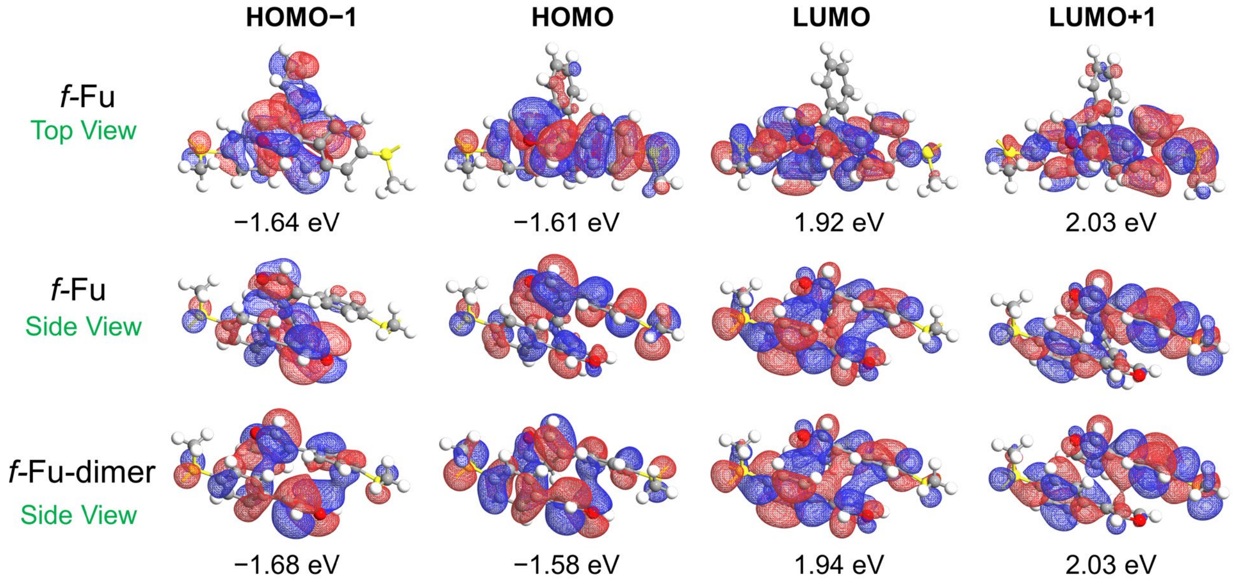

Supplementary Figure 36 MPSH of *f*-Fu and *f*-Fu-dimer. The charge distribution of HOMO–1, HOMO, LUMO and LUMO+1 for *f*-Fu from both top view and side view. *f*-Fu-dimer refers to the dimer model of *f*-Fu without the benzene holder, maintaining the same stacking geometry. The isovalue is 0.04.

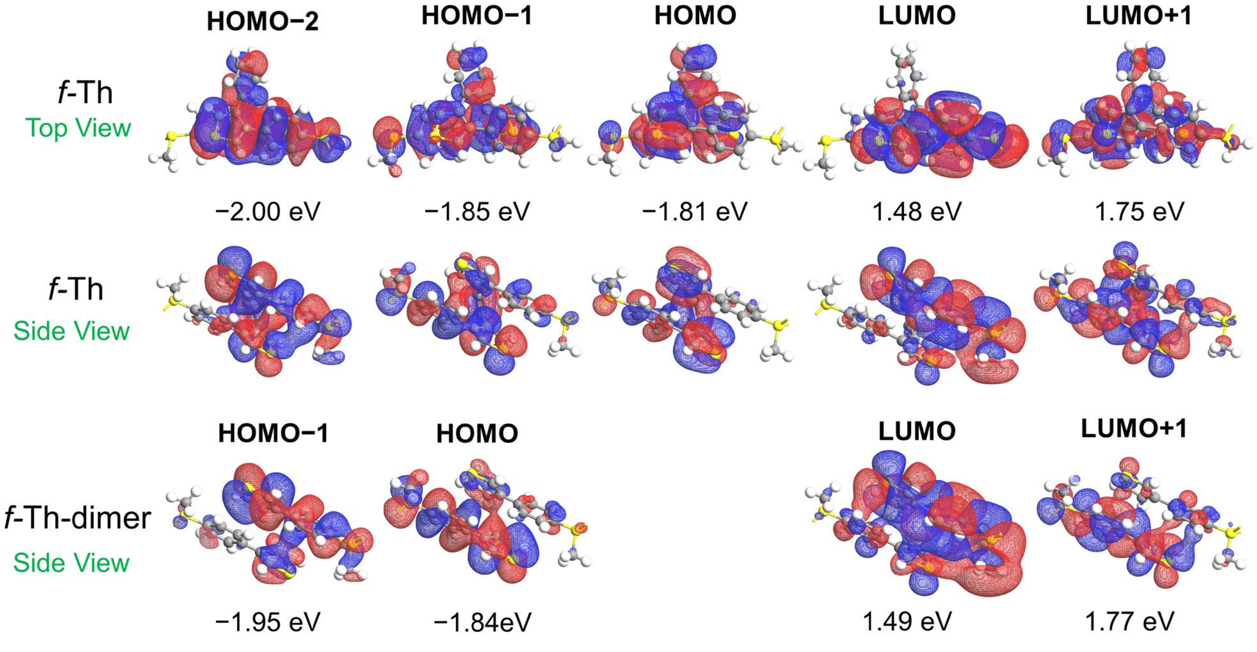

Supplementary Figure 37 MPSH of *f*-Th and *f*-Th-dimer. The charge distribution of HOMO–2, HOMO–1, HOMO, LUMO and LUMO+1 for *f*-Th from both top view and side view. *f*-Th-dimer refers to the dimer model of *f*-Th without the benzene holder, maintaining the same stacking geometry. The isovalue is 0.04.

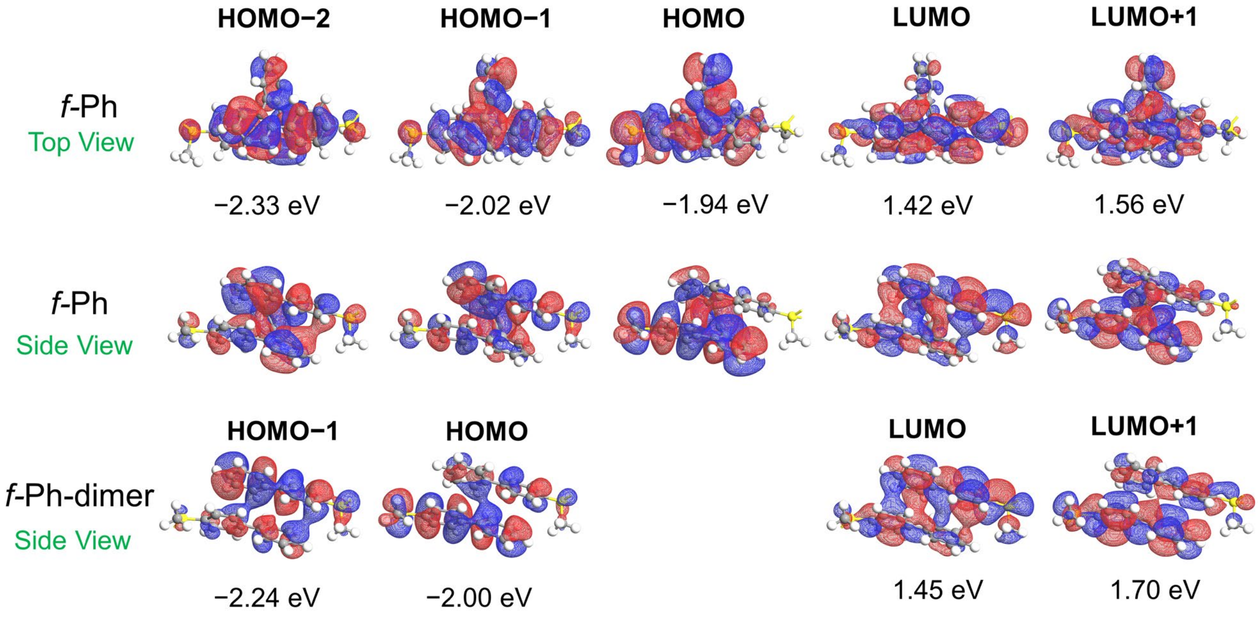

Supplementary Figure 38 MPSH of *f*-Ph and *f*-Ph-dimer. The charge distribution of HOMO–2, HOMO–1, HOMO, LUMO and LUMO+1 for *f*-Ph from both top view and side view. *f*-Ph-dimer refers to the dimer model of *f*-Ph without the benzene holder, maintaining the same stacking geometry. The isovalue is 0.04.

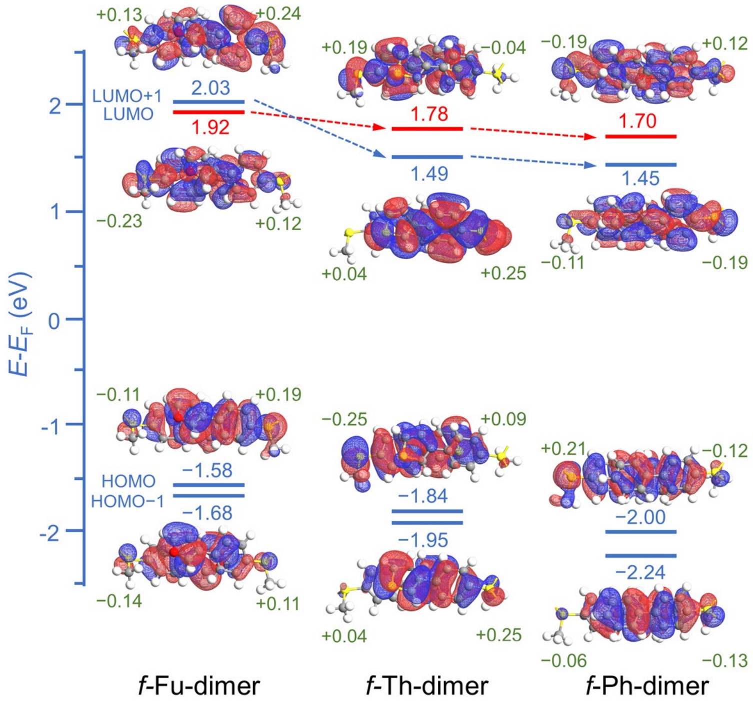

Supplementary Figure 39 The MPSHs and energy levels for *f*-34Th and *f*-34Fu. The orbital amplitudes of sulfur anchoring atoms are also labelled. The isovalue is 0.01 for LUMO and LUMO+1 for *f*-Th and the isovalue of the others is 0.04.

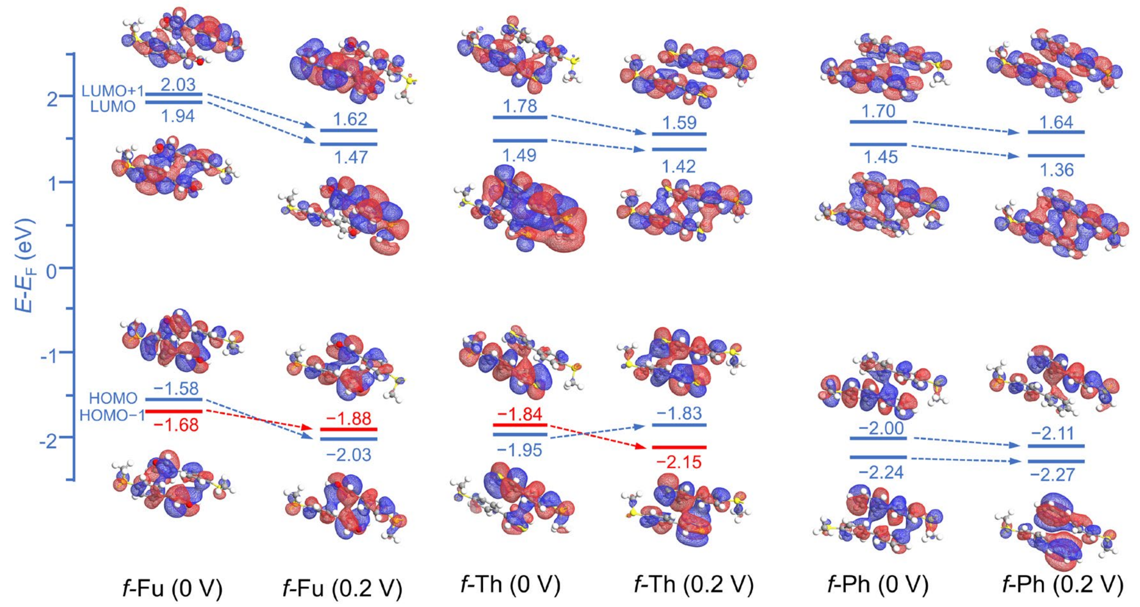

Supplementary Fig. 40 The MOs analyses with simplified dimers' models of *f*-Fu, *f*-Th and *f*-Ph at 0 V and 0.2 V. The isovalue is 0.04.

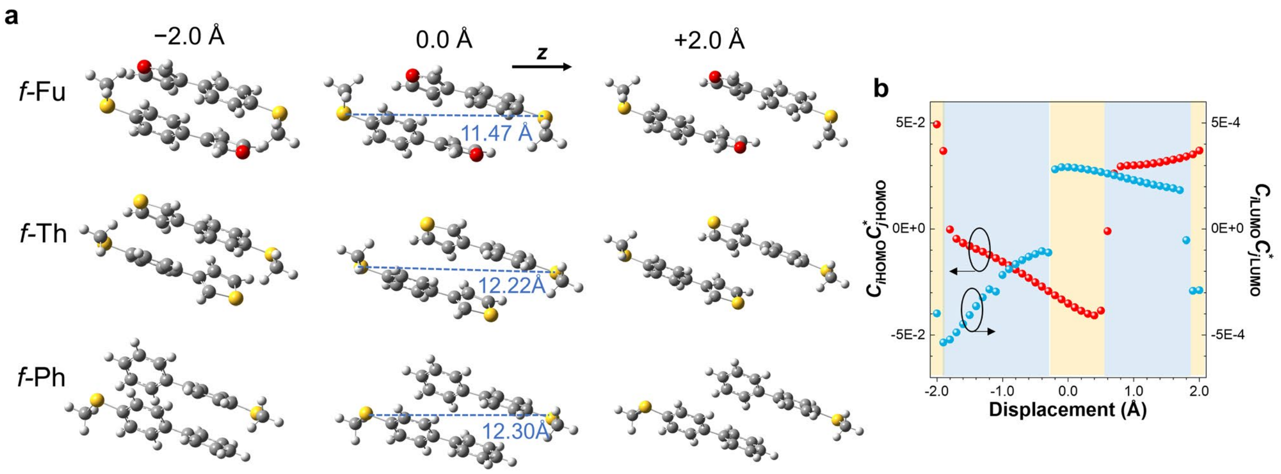

Supplementary Figure 41 Calculated HOMO and LUMO coupling based on dimer models. (**a**) The dimer models for coupling calculation. (**b**) The HOMO and LUMO coupling values of stacking arms with different displacement for *f*-Fu-dimer and *f*-Th-dimer. The zero-point is set as the stacking arms deconstructed from optimized junction configurations of foldamers.

Supplementary Note 4. Electrochemical gating experiment

The nature of electrochemical control in two-electrode electrochemical gating experiment based on STM-BJ is to form an asymmetric, bias-dependent electric double layer in polar environment to adjust the relative alignment between the Fermi level and molecular levels.[7,8] In polar solution with transferable ions, electric double layers at the tip and substrate are formed by ions' movement to screen out the electric field due to charges on the metal, influencing the electrostatic environment around the junction. Specifically, the coated tip is capable of the formation of a denser double layer with almost single-metal-atom exposition to the solvent, resulting in electrostatic asymmetry and bias polarity-dependent shift of the molecular resonance energy.

To understand the alteration of switching ratio between constructive quantum interference (CQI) and destructive quantum interference (DQI) transmission, Landauer-like expression is adopted as the description of current:

$$I = \frac{2e}{h}\int_{-eV/2}^{eV/2} T(E + \alpha eV)dE \qquad (2)$$

where e is the fundamental unit of charge, h is Planck's constant, V is the applied voltage, α is a coefficient describing the impact of the applied voltage on the resonance position analogous to a first-order Stark shift and $T(E + \alpha eV)$ is the energy-dependent transmission function for the junction. Here, α is set as 0.5. Given $G = I/V$, the conductance is directly proportional to the integral area of transmission. Considering CQI and DQI transmission in our foldamer system is independent to each other in break-junction measurement, the switching ratio between CQI and DQI transmission can be expressed as:

$$f = \frac{G_{CQI}}{G_{DQI}} = \frac{I_{CQI}}{I_{DQI}} \propto (S_{CQI} - S_{DQI}) \qquad (3)$$

where f refers to switching ratio and $S_{CQI/DQI}$ refers to the integral area of CQI/DQI transmission, and the difference value is shown as colored area in Supplementary Fig. 39. For foldamer system in this work, this model with a series of energy level diagrams is illustrated in Supplementary Fig. 39. At zero bias, the resonance is located at an energy ε relative to both the tip and substrate chemical potential. When a positive or negative (Supplementary Fig. 39e) voltage V is applied to the tip relative to the substrate, the bias window will open symmetrically. However, due to the tip-substrate asymmetry of the double layer, the resonance shifts by an amount equal to αeV towards the tip chemical potential. Here, α is set as 0.5. In polar media, when the tip is biased negatively, the molecular resonance is at $\varepsilon - eV$ relative to the tip chemical potential and at ε relative to the substrate chemical potential (Supplementary Fig. 39b). For this system, a large area of the CQI resonance and a small area of the DQI anti-resonance fall within the bias window and the difference (red shade) is distinct, suggesting higher switching ratio. When the tip is biased positively, the molecular resonance again remains pinned to the substrate chemical potential, but is at ε + eV relative to the tip chemical potential (Supplementary Fig. 39c). DQI antiresonance shifts away from the Fermi level, and the resonance of HOMO shifts towards the Fermi level, resulting in lower switching ratio. In non-polar solution, the resonance does not shift in response to the applied bias, while both tip and substrate chemical potentials shift relative to the resonance position. The area under the resonance that falls within the bias window is independent of the bias polarity (Supplementary Fig. 39d and 39e).

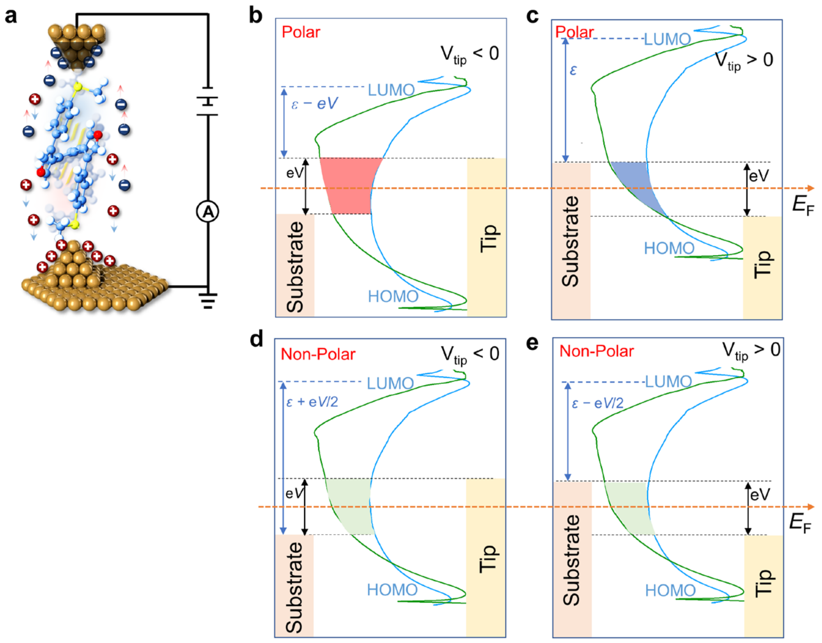

Supplementary Figure 42 Schematic illustration of the mechanism and experimental configuration of two-electrode electrochemical measurement. (**a**) Schematic illustration of two-electrode electrochemical gating measurement in PC with Apizeon wax coated gold tip. Energy level diagram and molecular transmission when the tip is biased negatively (**b**) and (**c**) relative to the substrate in polar media. Energy level diagram and molecular transmission when the tip is biased negatively (**d**) and positively (**e**) relative to the substrate in non-polar media.

Cyclic voltammetry (CV) *ex situ* was performed on a BAS 100W Bioanalytical Systems at a scan rate of 100 mV s^{-1}. Glassy carbon was used as the working electrode, platinum wire as the counter electrode and silver wire as a pseudo-reference electrode. The reduction was carried out in DMF solution and the oxidation was carried out in CH_2Cl_2 solution. The solutions were prepared at a concentration of 1 mM with 100 mM tetrabutylammonium hexafluorophosphate ($TBAPF_6$) as the electrolyte. All the data were referenced to the halfwave potential of the ferrocene/ferrocenium redox couple.

CV adopting the break-junction geometry was performed in a solution of propylene carbonate (PC) with 50 mM $TBAPF_6$ as supporting electrolyte. A coated Au tip serves as working electrode while a large gold substrate serves as counter electrode. The scan rate was 100 mV s^{-1}. The results show that within the biases' range from −1.5 V to 1.0 V, there is no Faraday process happening, indicative of no redox reaction.

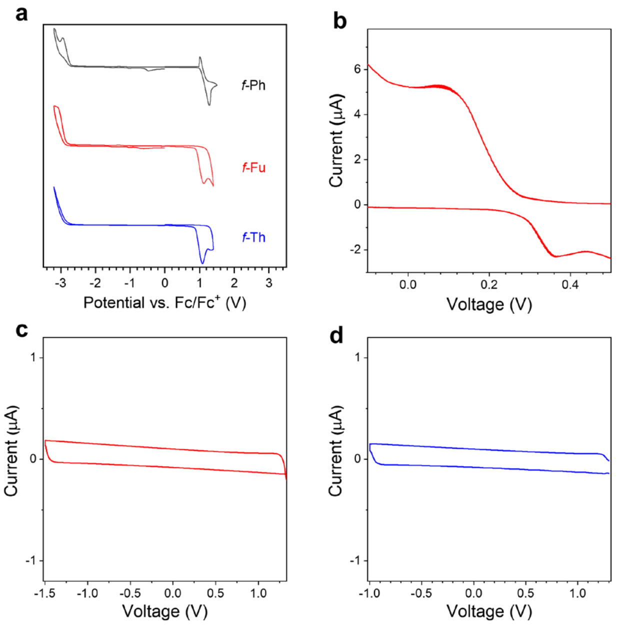

Supplementary Figure 43 Electrochemical measurement for foldamers. (**a**) The redox properties of *f*-Fu, *f*-Th and *f*-Ph. Cyclic voltammetry of reduction and oxidation are referenced to the Fc/Fc^+ redox couple. The reduction is processed in dimethylformamide and the oxidation in dichloromethane. (**b**) Cyclic voltammogram using the break-junction geometry. CV taken with solution of 1 mM ferrocene in PC with 50 mM $TBAPF_6$. (**c**) CV taken with solution of 0.1 mM *f*-Fu in PC with 50 mM $TBAPF_6$. (**d**) CV taken with solution of 0.1 mM *f*-Th in PC with 50 mM $TBAPF_6$.

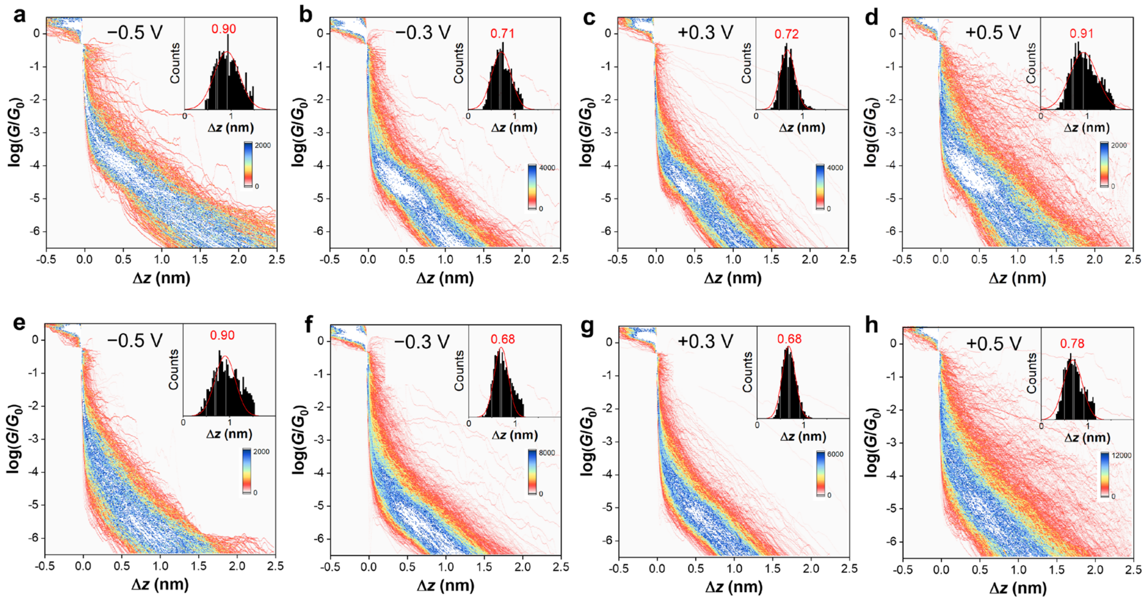

Supplementary Figure 44 Two-electrode gating experiment for *f*-Fu. The 0.1 mM PC solution is with 50 mM $TBAPF_6$. 2D histograms for the HC states of *f*-Fu under (**a**) –0.5 V, (**b**) –0.3 V, (**c**) +0.3 V and (**d**) +0.5 V. 2D histograms for the LC states of *f*-Fu under (**e**) –0.5 V, (**f**) –0.3 V, (**g**) +0.3 V and (**h**) +0.5 V.

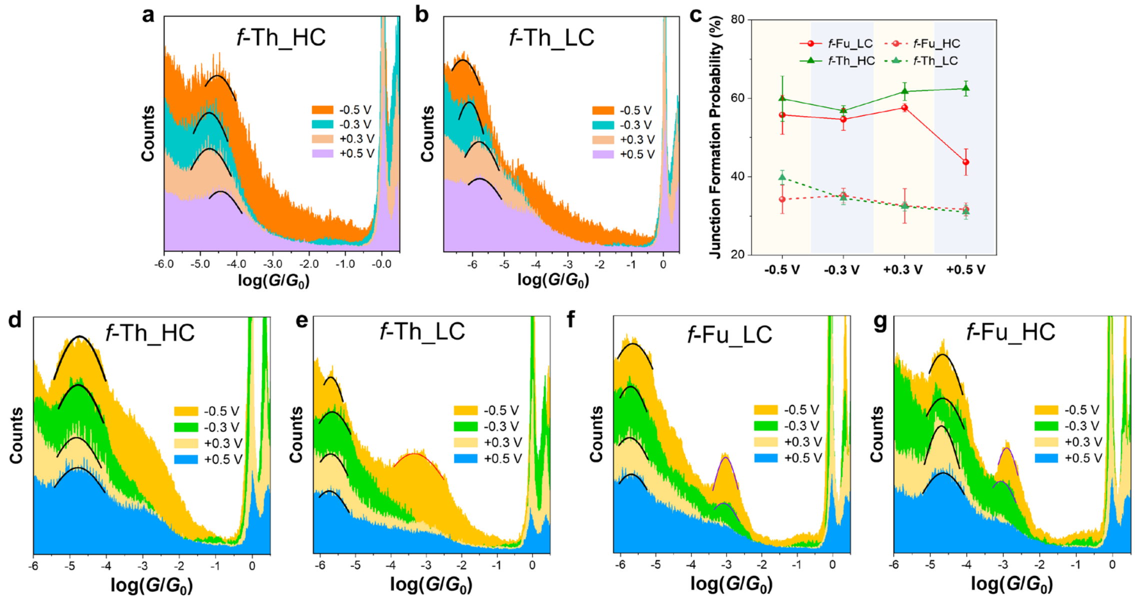

Supplementary Figure 45 The comparison for two-electrode gating experiment in ionic environment and regular conductance measurement in THF and TMB mixture. 1D histograms of (**a**) HC and (**b**) LC states of *f*-Th performed by two-electrode electrochemical gating experiments at different biases. **c** Statistic junction formation probabilities of *f*-Fu and *f*-Th under different applied biases in PC with 50 mM $TBAPF_6$. The error bars are the standard deviation of multiple results for junction formation probabilities in conductance measurment over three times. 1D histograms of (**d**) HC and (**e**) LC states of *f*-Th performed by regular conductance measurement in THF: TMB (1:4, *v*/v) at different biases. 1D histograms of (**f**) LC and (**g**) HC states of *f*-Fu performed by regular conductance measurement in THF: TMB (1:4, *v*/v) at different biases.

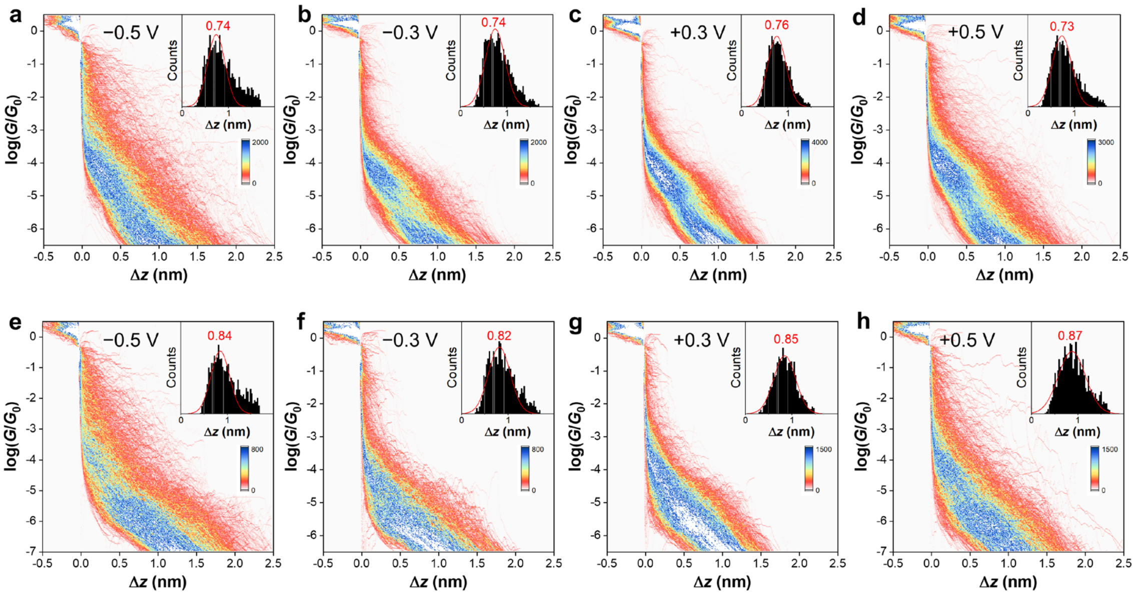

Supplementary Figure 46 Two-electrode gating experiment for *f*-Th. The 0.1 mM PC solution is with 50 mM $TBAPF_6$. 2D histograms for the HC states of *f*-Th under (**a**) –0.5 V, (**b**) –0.3 V, (**c**) +0.3 V and (**d**) +0.5 V. 2D histograms for the LC states of *f*-Th under (**e**) –0.5 V, (**f**) –0.3 V, (**g**) +0.3 V and (**h**) +0.5 V.

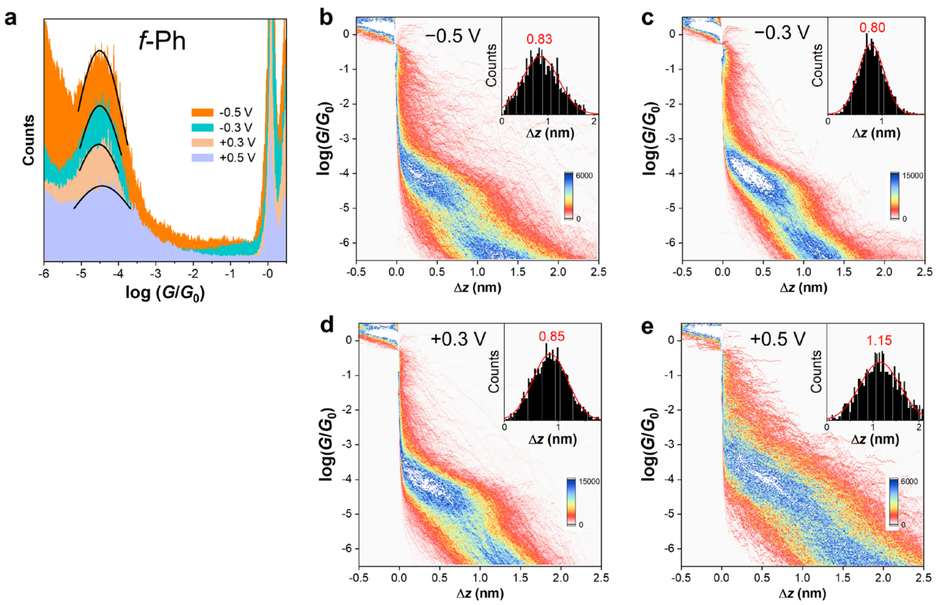

Supplementary Figure 47 Two-electrode gating experiment for *f*-Ph. The 0.1 mM PC solution is with 50 mM $TBAPF_6$. (**a**) 1D histograms for *f*-Ph under different biases. 2D histograms for *f*-Ph under (**b**) –0.5 V, (**c**) –0.3 V, (**d**) +0.3 V and (**e**) +0.5 V.

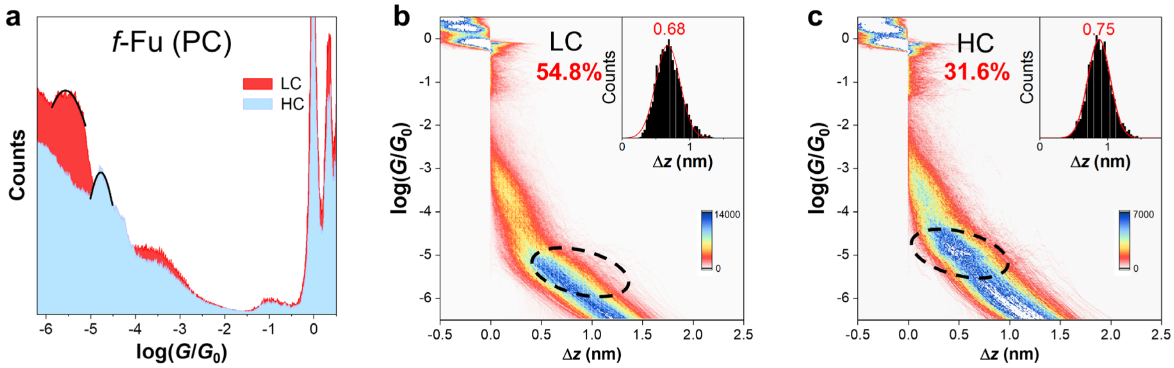

Supplementary Figure 48 Conductance measurement for *f*-Fu in PC. (**a**) 1D histograms of HC and LC states of *f*-Fu in PC at 0.1 V. 2D conductance–displacement histograms of HC states (**b**) and LC states (**c**) for *f*-Fu in PC with inserted relative displacement distribution histogram.

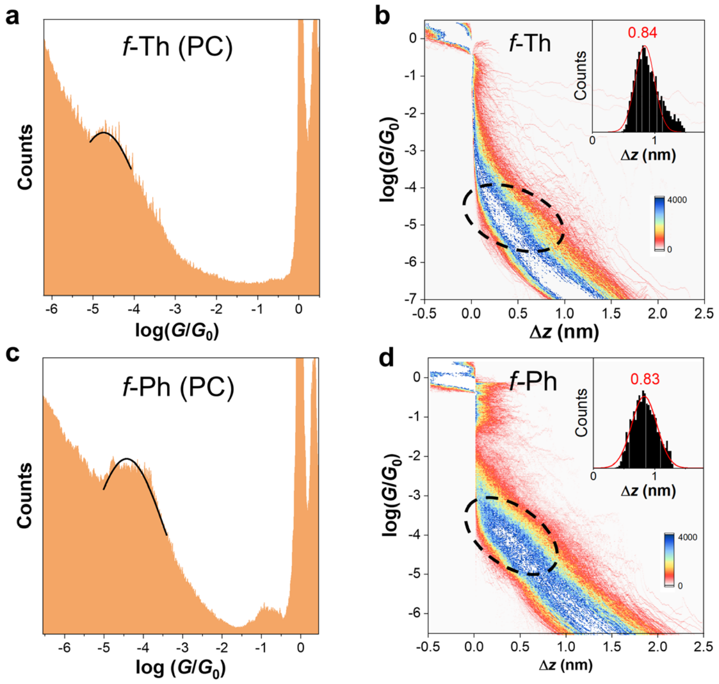

Supplementary Figure 49 Conductance measurement for *f*-Th and *f*-Ph in PC. (**a**) 1D histograms of *f*-Th in PC at 0.1 V. (**b**) 2D conductance–displacement histograms of *f*-Th in PC with inserted relative displacement distribution histogram. (**c**) 1D histograms of *f*-Ph in PC at 0.1 V. (**d**) 2D conductance–displacement histograms of *f*-Ph in PC with inserted relative displacement distribution histogram.

Supplementary Note 5. NMR spectra

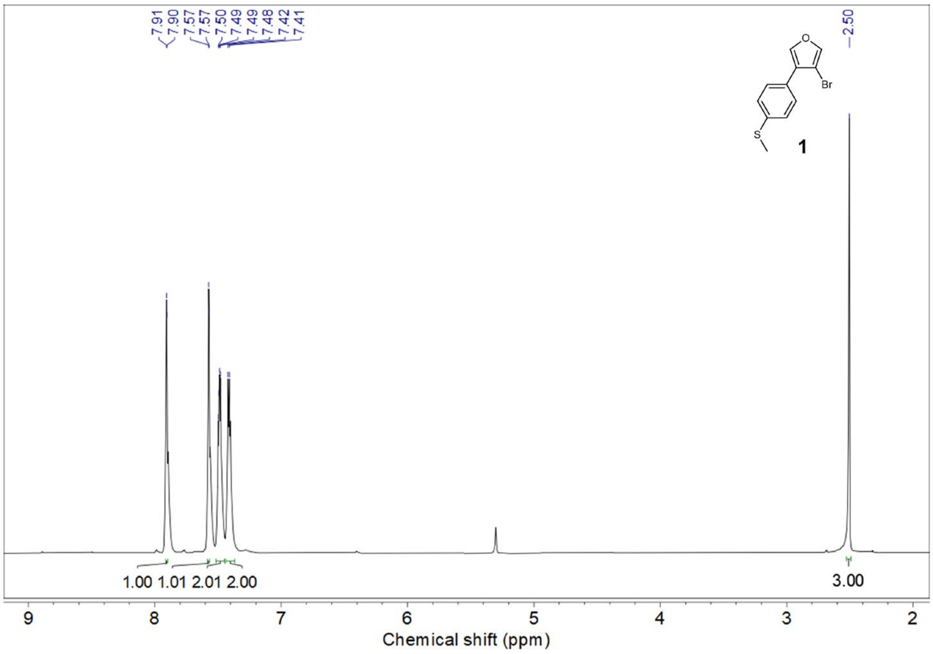

Supplementary Figure 50 ^{1}H NMR spectrum (500 MHz, CD_2Cl_2, 298 K) of molecule **1**.

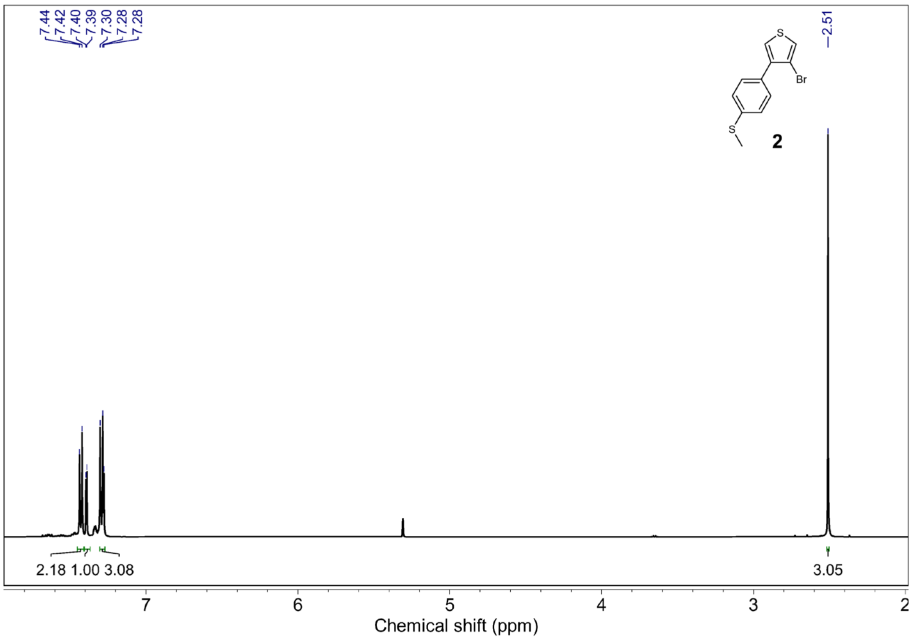

Supplementary Figure 51 ^{1}H NMR spectrum (500 MHz, CD_2Cl_2, 298 K) of molecule **2**.

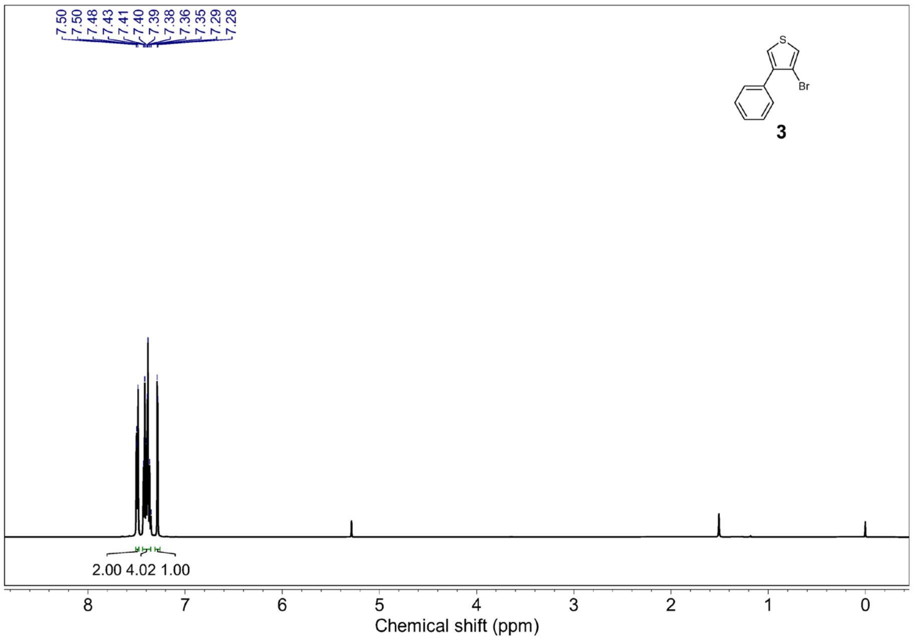

Supplementary Figure 52 ^{1}H NMR spectrum (500 MHz, CD_2Cl_2, 298 K) of molecule **3**.

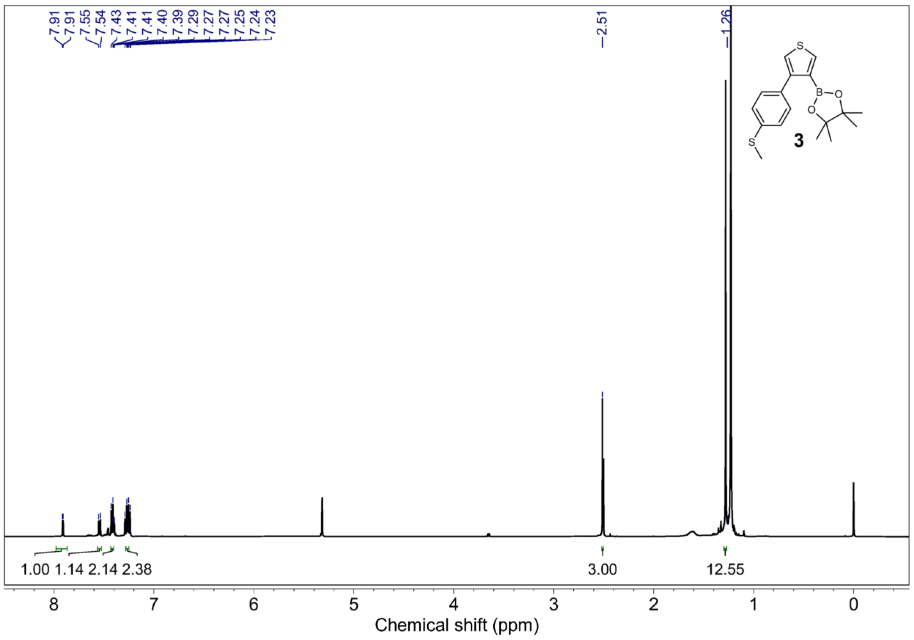

Supplementary Figure 53 ^{1}H NMR spectrum (500 MHz, CD_2Cl_2, 298 K) of molecule **4**.

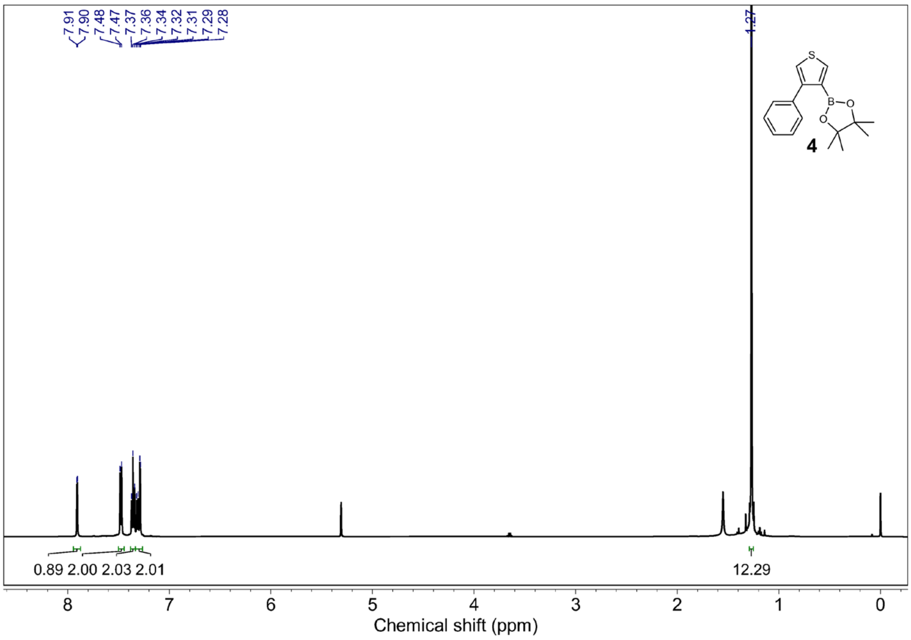

Supplementary Figure 54 ^{1}H NMR spectrum (500 MHz, CD_2Cl_2, 298 K) of molecule **5**.

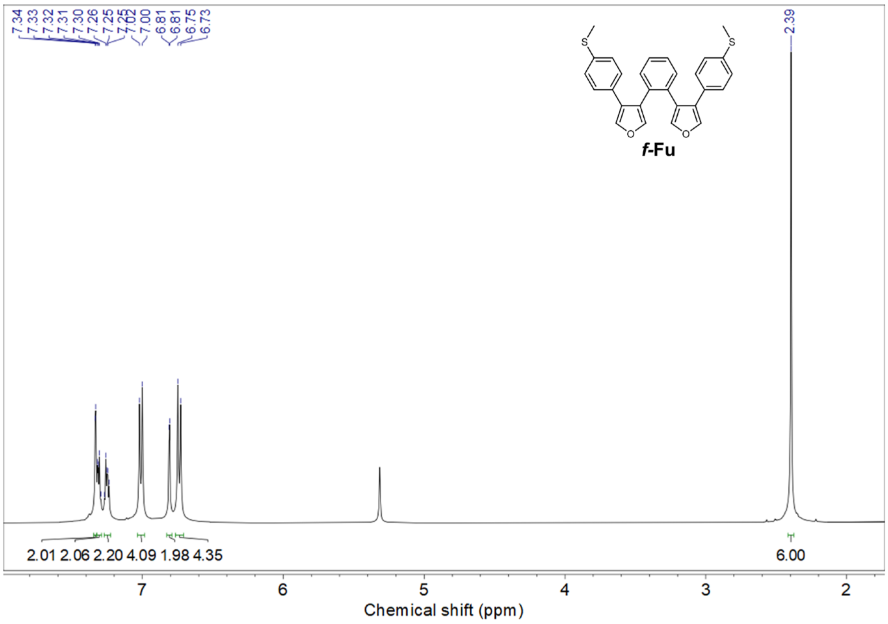

Supplementary Figure 55 ^{1}H NMR spectrum (500 MHz, CD_2Cl_2, 298 K) of *f*-Fu.

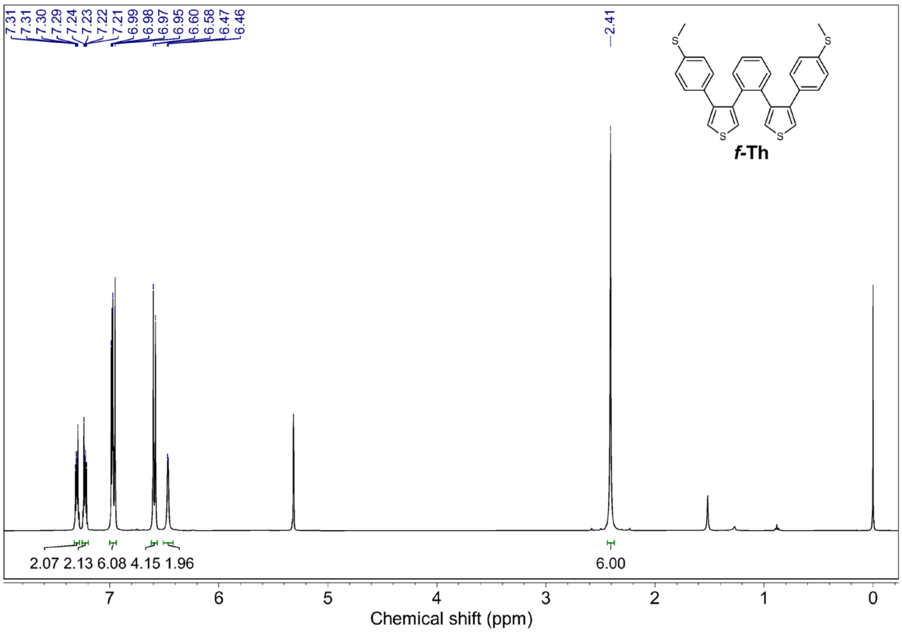

Supplementary Figure 56 ¹H NMR spectrum (500 MHz, CD_2Cl_2, 298 K) of *f*-Th.

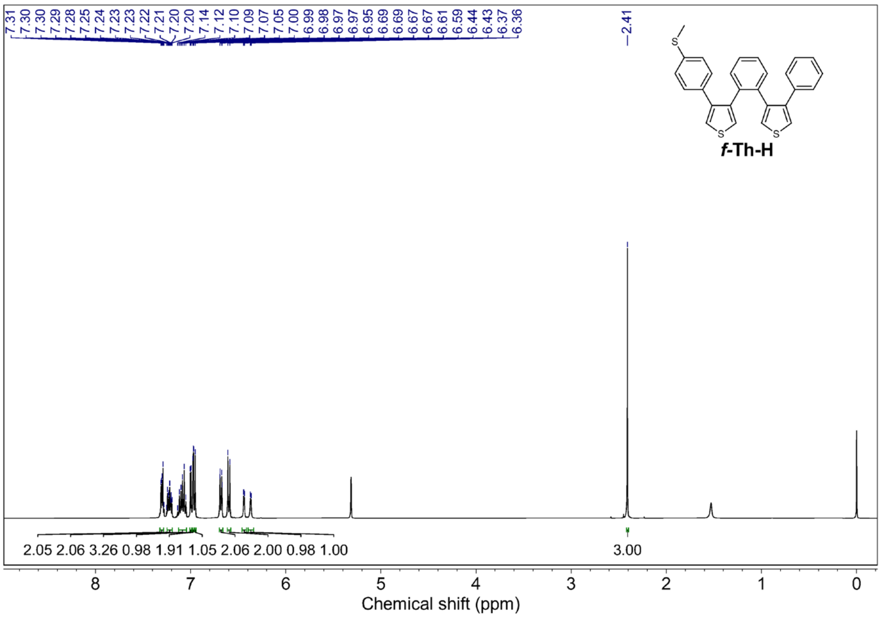

Supplementary Figure 57 ¹H NMR spectrum (500 MHz, CD_2Cl_2, 298 K) of *f*-Th.

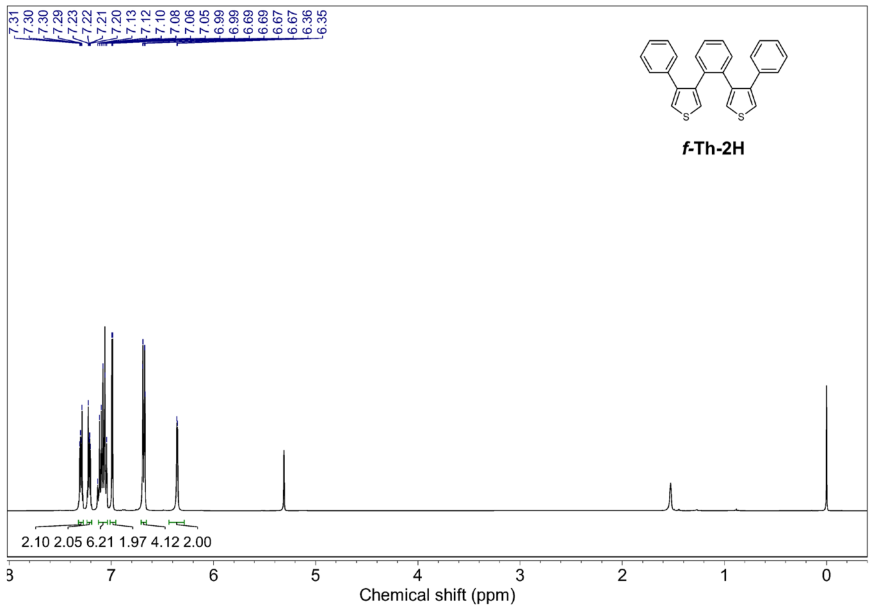

Supplementary Figure 58 ^{1}H NMR spectrum (500 MHz, CD_2Cl_2, 298 K) of *f*-Th-2H.

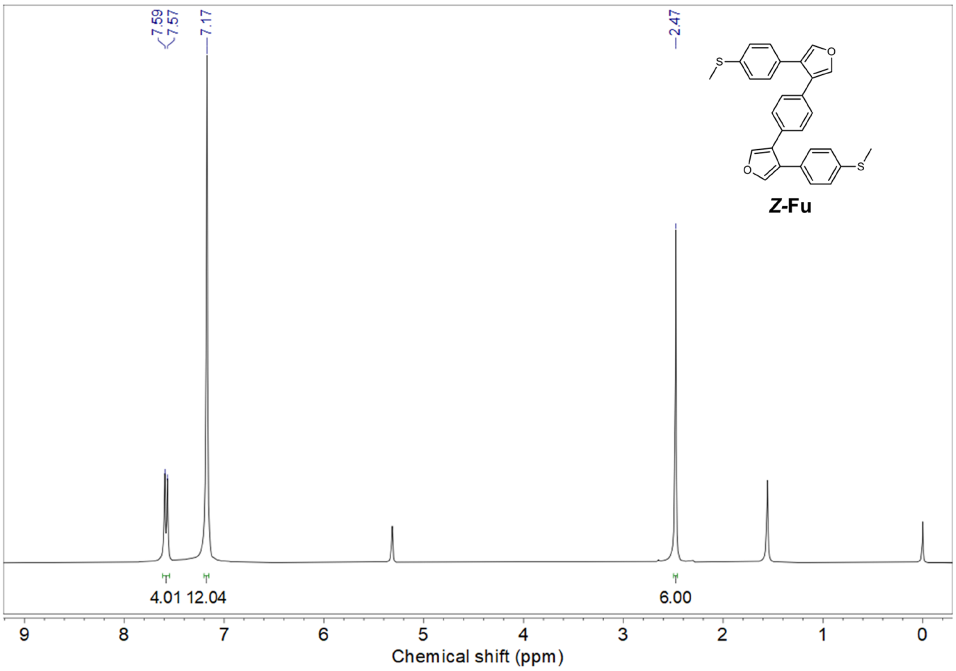

Supplementary Figure 59 ^{1}H NMR spectrum (500 MHz, CD_2Cl_2, 298 K) of *Z*-Fu.

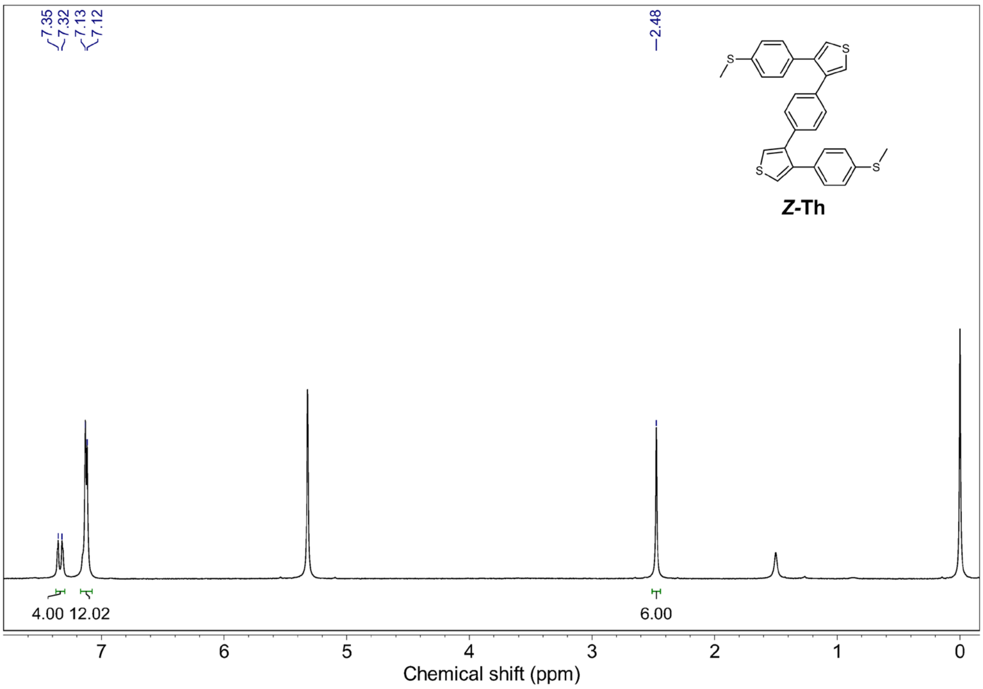

Supplementary Figure 60 ^{1}H NMR spectrum (500 MHz, CD_2Cl_2, 298 K) of *Z*-Th.

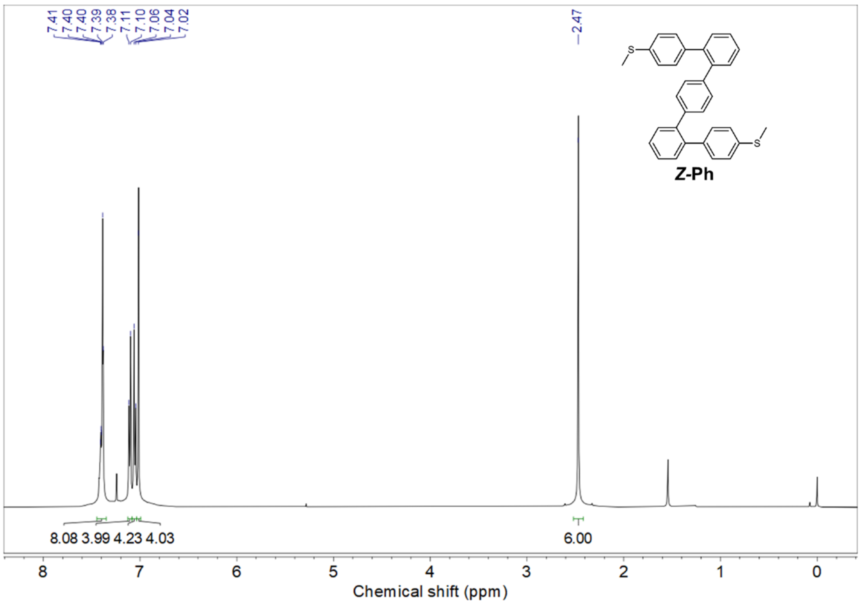

Supplementary Figure 61 ^{1}H NMR spectrum (500 MHz, CD_2Cl_2, 298 K) of *Z*-Ph.

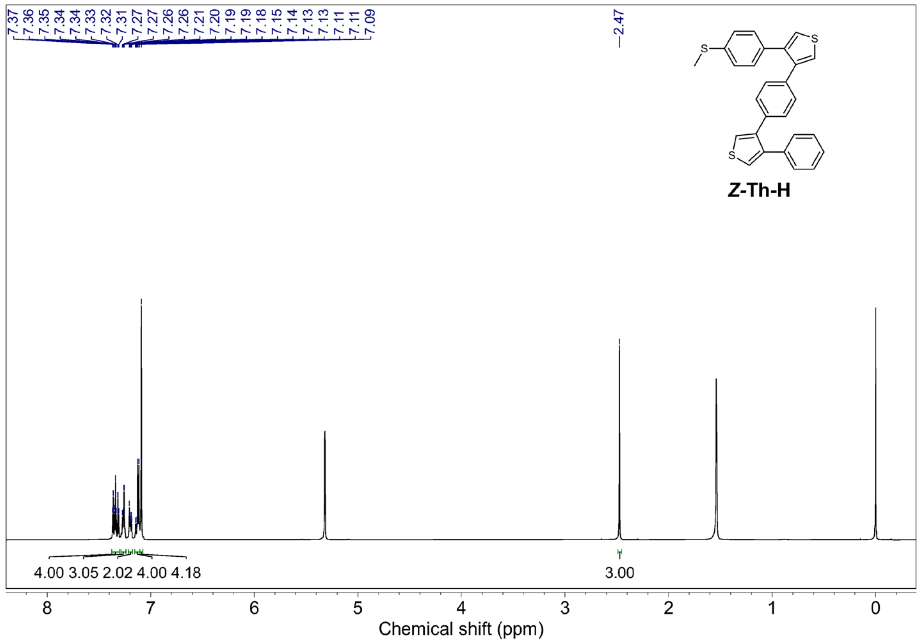

Supplementary Figure 62 ^{1}H NMR spectrum (500 MHz, CD_2Cl_2, 298 K) of *Z*-Th-H.

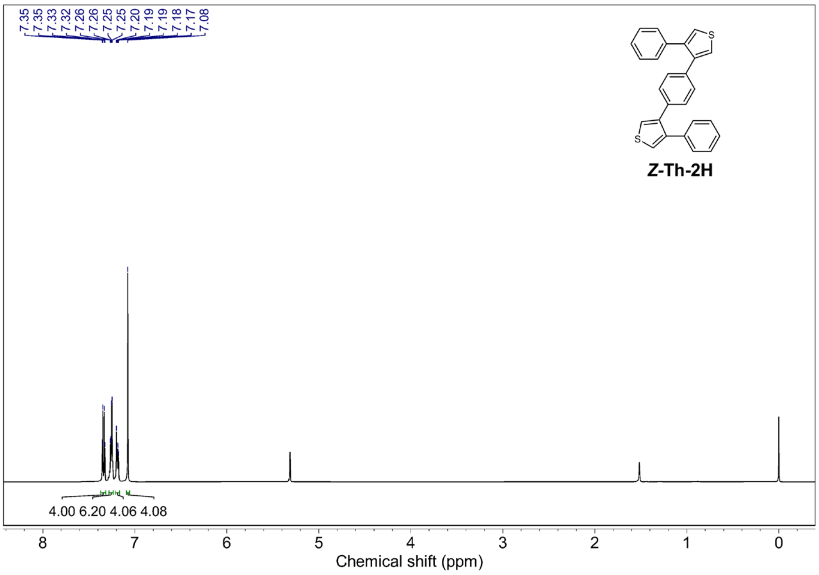

Supplementary Figure 63 ^{1}H NMR spectrum (500 MHz, CD_2Cl_2, 298 K) of *Z*-Th-2H.

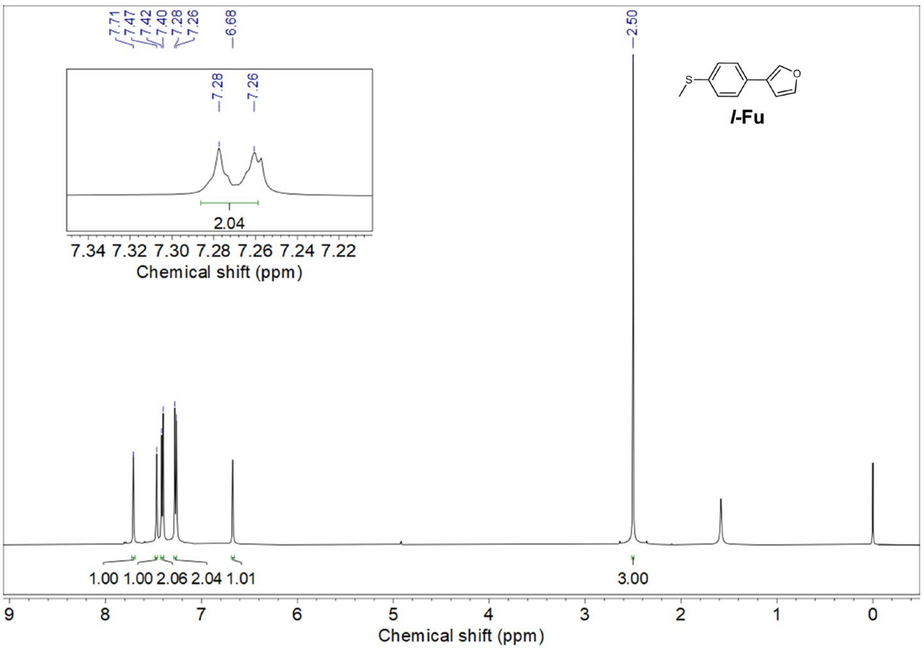

Supplementary Figure 64 ^{1}H NMR spectrum (500 MHz, CD_3Cl, 298 K) of *l*-Fu.

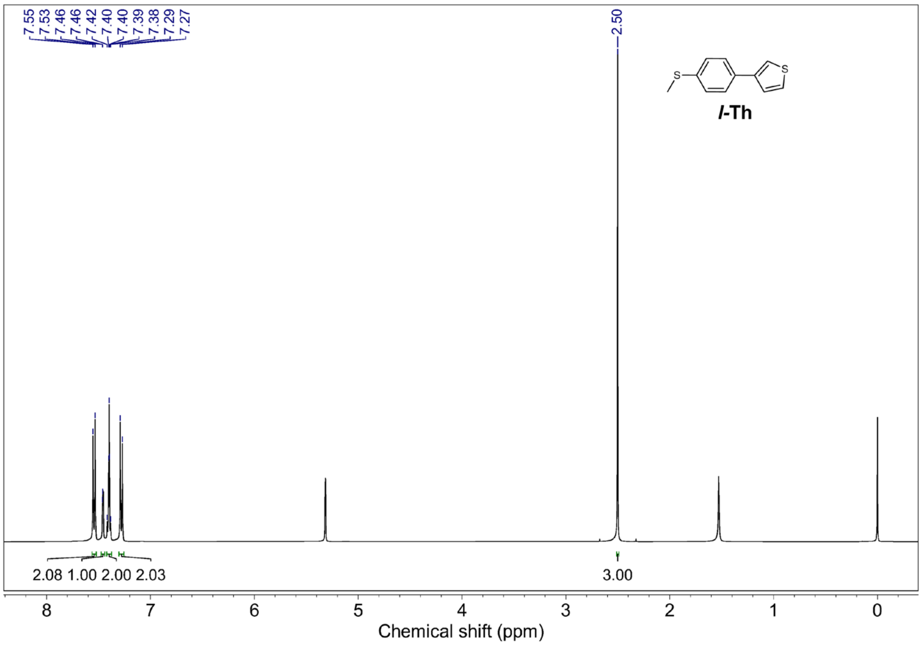

Supplementary Figure 65 ^{1}H NMR spectrum (500 MHz, CD_2Cl2, 298 K) of *l*-Th.

Supplementary Note 6. References

(1) Li, J., Shen, P., Zhen, S., Tang, C., Ye, Y., Zhou, D., Hong, W., Zhao, Z. & Tang, B. Z. Mechanical single-molecule potentiometers with large switching factors from *ortho*-pentaphenylene foldamers. *Nat. Commun*. **12**, 167(2021).

(2) Hong, W., Valkenier, H., Meszaros, G., Manrique, D. Z., Mishchenko, A., Putz, A., Garcia, P. M., Lambert, C. J., Hummelen, J. C. & Wandlowski, T. An MCBJ case study: The influence of π-conjugation on the single-molecule conductance at a solid/liquid interface. *Beilstein. J. Nanotechnol.* **2**, 699–713(2011).

(3) Hong, W., Manrique, D. Z., Moreno-Garcia, P., Gulcur, M., Mishchenko, A., Lambert, C. J., Bryce, M. R. & Wandlowski, T. Single molecular conductance of tolanes: experimental and theoretical study on the junction evolution dependent on the anchoring group. *J. Am. Chem. Soc.* **134**, 2292–2304(2012).

(4) Li, X. *et al.* Structure-Independent conductance of thiophene-based single stacking junctions. *Angew. Chem. Int. Ed.* **59**, 3280−3286(2019).

(5) Lu, T. & Chen, F. Multifwn: A multifunctional wavefunction analyzer. *J. Comput. Chem.* **33**, 580–592(2012).

(6) Taylor, J. Brandbyge, M. & Stokbro, K. Theory of rectification in tour wires: The role of electrode coupling. *Phys. Rev. Lett*. **89**, 138301(2002).

(7) Greenwald, J. E., Cameron, J., Findlay, N. J., Fu, T., Gunasekaran, S., Skabara, P. J. & Venkataraman, L. Highly nonlinear transport across single-molecule junctions via destructive quantum interference. *Nat. Nanotechnol.* **16**, 313−317(2021).

(8) Capozzi, B., Xia, J., Adak, O., Dell, E. J., Liu, Z. F., Taylor, J. C., Neaton, J. B., Campos, L. M. & Venkataraman, L. Single-molecule diodes with high rectification ratios through environmental control. *Nat. Nanotechnol*. **10**, 522−527(2015).
